# A Flow Cytometric Method to Quantify the Endosomal Escape of a Protein Toxin to the Cytosol of Target Cells

**DOI:** 10.1007/s11095-019-2725-1

**Published:** 2019-12-23

**Authors:** Harrison J. Wensley, David A. Johnston, Wendy S. Smith, Suzanne E. Holmes, Sopsamorn U. Flavell, David J. Flavell

**Affiliations:** 1 The Simon Flavell Leukaemia Research Laboratory, Southampton General Hospital, Southampton, SO16 6YD UK; 2 Clinical and Experimental Sciences, University of Southampton Faculty of Medicine, Southampton General Hospital, Southampton, SO16 6YD UK; 3 Biomedical Imaging Unit, University of Southampton Faculty of Medicine, Southampton General Hospital, Southampton, SO16 6YD UK; 4 University of Southampton Faculty of Medicine, Southampton General Hospital, Southampton, SO16 6YD UK

**Keywords:** Endosomal escape, Flow cytometry, Immunotoxin, Saponin, Saporin

## Abstract

**Purpose:**

The aim of this work was to develop a quantitative, flow cytometric method for tracking the endolysosomal escape of a fluorescently labelled saporin toxin.

**Methods:**

Flow cytometric measurements of fluorescent pulse width and height were used to track the endocytic uptake into Daudi cells of a fluorescently labelled saporin toxin and the saporin based immunotoxin, OKT10-SAP. Subsequently, measurement of changes in pulse width were used to investigate the effect of a triterpenoid saponin on the endolysosomal escape of internalised toxin into the cytosol. Live cell confocal microscopy was used to validate the flow cytometry data.

**Results:**

Increased endolysosomal escape of saporin and OKT10-SAP was observed by confocal microscopy in cells treated with saponin. Fluorescent pulse width measurements were also able to detect and quantify escape more sensitively than confocal microscopy. Saponin induced endolysosomal escape could be abrogated by treatment with chloroquine, an inhibitor of endolysosomal acidification. Chloroquine abrogation of escape was also mirrored by a concomitant abrogation of cytotoxicity.

**Conclusions:**

Poor endolysosomal escape is often a rate limiting step for the cytosolic delivery of protein toxins and other macromolecules. Pulse width analysis offers a simple method to semi-quantify the endolysosomal escape of this and similar molecules into the cytosol.

**Electronic supplementary material:**

The online version of this article (10.1007/s11095-019-2725-1) contains supplementary material, which is available to authorized users.

## Introduction

The delivery of macromolecules, such as protein therapeutics or nucleic acids, into cells often depends on endocytic processes. Following internalisation into the early endosome, the compartment matures into the late endosome before finally fusing with lysosomes to form the endo-lysosome where the contents are degraded by lysosomal enzymes [1]. Therefore, for these macromolecules to reach their targets in the cytosol, they must escape the endolysosomal compartment before they are degraded. In order to combat this problem, considerable research has been undertaken to identify carrier molecules and other pharmacological agents able to enhance this escape. A reliable and sensitive assay to measure this endosomal release would be a valuable tool in the development of such therapeutics. We present here a flow cytometric assay to measure the endolysosomal escape of the type I ribosome inactivating protein saporin, a key component of many targeted toxins [2–4].

Targeted toxins consist of a protein toxin with enzymatic activity coupled to a tumour specific targeting domain such as a cytokine, growth factor or antibody (immunotoxin). These chimeric molecules represent an opportunity for a highly specific cancer therapy. The majority of targeted toxins incorporate either bacterial toxins such as pseudomonas exotoxin or diphtheria toxin, or plant derived ribosome inactivating toxins such as saporin or ricin A chain. All of these toxins act to irreversibly arrest protein synthesis but must first gain access to the cytosol to reach their intracellular targets [5–7]. Whilst coupling the toxin to a targeting domain offers specificity, it does not guarantee clinical efficacy. Several obstacles to the efficient delivery of toxins into the cytosol have been identified. These include but are not restricted to: poor internalisation of the immunotoxin [8], recycling of the immunotoxin back to the cell surface [9] and poor endosomal escape to the cytosol resulting in trafficking of the toxin component to the lysosome and subsequent degradation [10,11].

A number of approaches have been used to improve the efficiency of endolysosomal escape of toxins into the cytosol. Examples include chemical enhancers such as the lysosomotropic amines and carboxylic ionophores [12,13]. These act by raising the lysosomal pH thereby inhibiting the degradation of internalised toxins by pH dependent lysosomal enzymes. This is in contrast to viral and bacterial cell penetrating peptides which directly enable endosomal escape [14]. One family of promising augmentative agents are the saponins, a group of membrane permeabilising plant derived glycosides [15]. Previous work has shown that saponins appear to enable the endolysosomal escape of saporin and saporin derived immunotoxins, but the mechanism by which this occurs is not yet fully elucidated [16–18].

Currently, the efficacy of endosomal escape enhancers is evaluated either indirectly through measurement of changes to the cytotoxic activity of the toxin or through microscopic imaging of fluorescently labelled toxins. Confocal fluorescence microscopy can provide specific information on the intracellular location of labelled macromolecular species of interest. However, microscopic imaging has a low throughput and only provides information on a relatively small number of cells at a time. Furthermore, quantitative analysis of microscopic images is time consuming and subject to potential operator bias in cell selection.

**Fig. 1:**
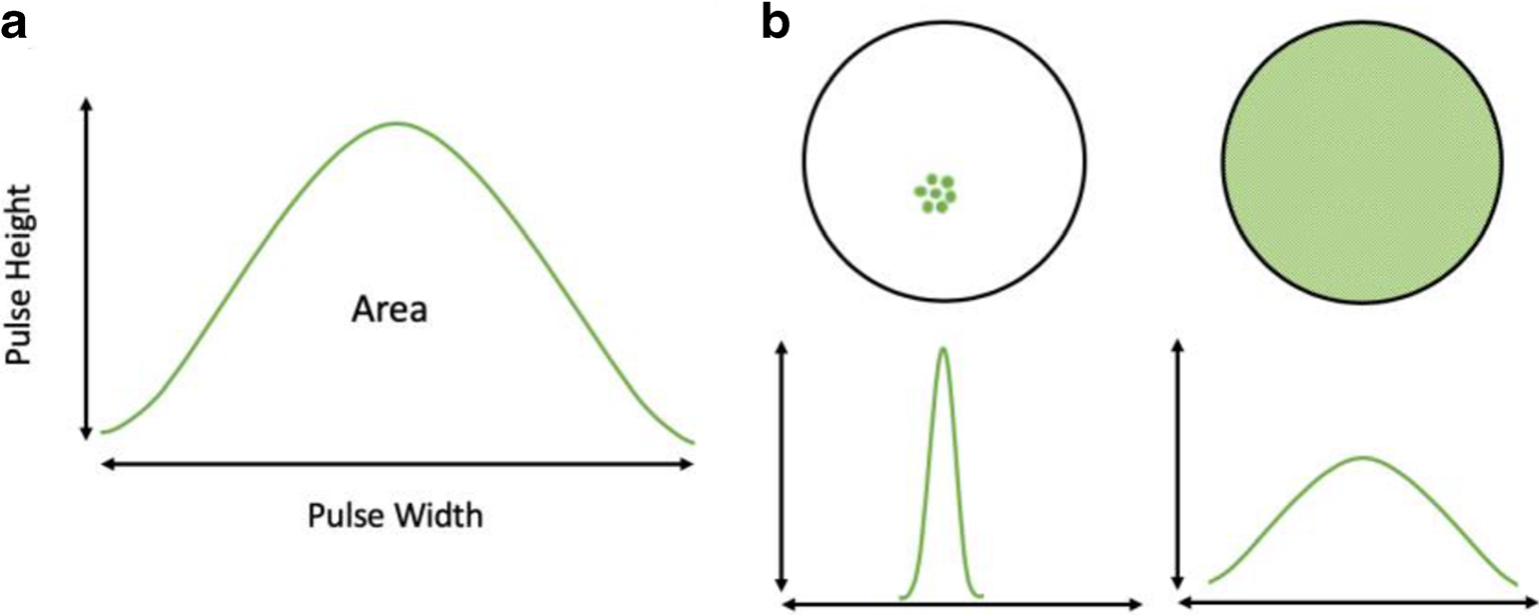
Pulse shape analysis. (a) Flow cytometry signal pulse for a fluorescent particle. The pulse height is the maximum fluorescence intensity. Pulse width represents the amount of time taken for the particle to pass through the laser. (b) The distribution of a fluorescent label within the cell changes the fluorescent pulse width. A localised, vesicular distribution will result in a narrow pulse width. In contrast, fluorescent label with a diffuse, cytosolic distribution will be recorded as a wide pulse width

Flow cytometry is capable of recording information from thousands of cells per second and is ideal for quantitative analysis of samples. The information for an entire cell is recorded as a signal pulse of voltage over time from which several parameters can be quantified. Typically, the most commonly reported parameter is the area under the pulse or the ‘pulse area’ which is proportional to the total fluorescence emitted from the cell. Two other important parameters that define the signal pulse are the pulse height and the pulse width. The pulse height represents the point of maximum fluorescence intensity within the cell whilst the pulse width provides information on the amount of time a fluorescent signal was detected as the cell passed through the laser beam. Pulse width can therefore be used to characterise the distribution of a fluorescent marker within the cell. Analysis of pulse width has been used to investigate nuclear enlargement [19], the aggregation states of cytoplasmic proteins [20] and to differentiate between surface bound and golgi located fluorescent markers [21,22]. In the context of measuring the efficacy of endolysosomal escape enhancers, pulse width analysis may provide a valuable method of differentiating cells where escape of a fluorescently labelled toxin has occurred. In cells where the toxin has accumulated purely within the endolysosomal compartment the corresponding voltage pulse should be tall and narrow consistent with a punctate distribution. Endosomal escape of the toxin and its subsequent diffusion throughout the cytoplasm would lead to an increase in the pulse width as exemplified in Fig. 1. Measurement of this change across a large number of cells would allow for the quantification of endolysosomal escape enhancer efficacy.

In this study we demonstrate the use of pulse width analysis as a tool to investigate the enhancement of the endolysosomal escape of saporin and the saporin based immunotoxin (IT) OKT10-SAP by triterpenoid saponins from Gypsophila species.

## Materials and Methods

### Materials

Chloroquine (C6628), RPMI 1640 (R0883), phenolphthalein-free RPMI 1640 (R7509), sodium pyruvate (S8636), glutamine (G7513), foetal calf serum (FCS) (F0926), 2,3-Bis-(2-methoxy-4-nitro-5-sulphophenyl)-2H-tetrazolium-5-carboxanilide (XTT), phenazine methosulfate (P9625), bisBenzimide Hoechst 33342 trihydochloride (B2261) and anti-EEA1 antibody produced in chicken (GW21443A) were obtained from Merck (Sigma-Aldrich Company Ltd., Gillingham, UK). Alexa-Fluor 488 tetrafluorophenyl ester (TFP) was from Life Technologies (Eugene, OR, USA). Anti-human LAMP-1/CD107a monoclonal mouse IgG2B (clone 508,921) was obtained from R + D Systems (Minneapolis, MN, USA). Goat anti-mouse IgG Alexa Fluor 568 (ab175473) was supplied by Abcam (Cambridge, UK). Goat anti-chicken IgY (H + L) Alexa Fluor 555 (A21437) came from Invitrogen (Thermofisher Scientific, Loughborough, UK).

### Cell Lines

The Daudi human Burkitt lymphoma cell line [23] and the T cell acute lymphoblastic leukaemia cell line HSB-2 [24] were obtained from the European Collection of Cell Cultures (ECACC, Porton Down, Salisbury, UK). Cell lines were authenticated using the Identifier Plus DNA profiling system (Applied Biosciences, Carlsbad, CA, USA). Working cell banks were produced and frozen in liquid nitrogen. Cultures were maintained in the logarithmic growth phase by regular passage in RPMI 1640 medium containing 10% foetal calf serum (FCS) and supplemented with 2 mM glutamine and 2 mM sodium pyruvate (referred to hereafter as R10) at 37°C in 7% CO_2_ in a humidified environment for no longer than four weeks following which a fresh vial of cells were resurrected from the working cell bank.

### Saponinum Album

Saponinum Album (SA), an extract of saponins from *Gypsophila paniculata* L. and *Gypsophila arrostii* Guss was a commercial preparation from Merck (Darmstadt, Germany). SA contains a mixture of saponin species with the same aglycone core but possessing varying carbohydrate side chains [25]. The structures of the most abundant of these, SA1641 and SA1657 have been described previously [25].

### Saporin

The SO6 isoform of saporin was extracted and purified from the seeds of *Saponaria officinalis L*. (Soapwort) (Chiltern Seeds, Ulverston, Cumbria, UK) as described elsewhere [26].

### Immunotoxin

The IgG_1_ murine monoclonal antibody OKT10 against human CD38 was produced from cultures of the OKT10 hybridoma cell line [17]. OKT10 was covalently coupled to the SO6 isoform of saporin using the heterobifunctional cross-linking reagent SPDP as described previously [4]. The antibody:toxin ratios of the resulting conjugate, termed OKT10-SAP, were determined, following densitometric scanning bands shown in Fig. S1, to be, as a percentage of the total protein present: 1:1; ~55%; 1:2; ~10% and ~15% which could be either 1:3 or a 2:2 dimer. Alongside these conjugates there was also determined to be ~10% free antibody and ~10% free saporin.

### Fluorescent Labelling of Saporin and OKT10-SAP

To detect the trafficking of internalised saporin and OKT10-SAP together with their proposed endolysosomal escape in the presence of SA, fluorescent conjugates were constructed with an Alexa Fluor 488 5-TFP (Life Technologies, OR, USA). This was achieved by adding 800 μl of 9.3 mg/ml saporin SO6 or 3.5 mg/ml OKT10-SAPORIN to 100 μl carbonate buffer (1 M NaHCO_3_, pH 9.0) and 100 μl of Alexa Fluor 488 5-TFP (10 mg/ml in DMSO). Following stirring for 1 h at room temperature to effect conjugation, unconjugated fluorophore was removed by exhaustive dialysis for two hours at 4°C against 2 L PBS followed by a further 2 L of PBS overnight at 4°C. The concentrations of the resultant fluorescent conjugates were calculated using the Beer-Lambert law from their absorbance at 280 nm and 495 nm as measured on a Hitachi U1100 Spectrophotometer.

### Cell Culture

All experiments were conducted in phenolphthalein-free RPMI 1640 containing 10% FCS and supplemented with 2 mM glutamine and 2 mM sodium pyruvate.

### Endocytosis of SAP-AF and OKSAP-AF

Daudi and HSB-2 cells were incubated in 24-well plate wells with 1 × 10^−6^ M SAP-AF or 5 × 10^−8^ M OKSAP-AF in R10 at 37°C, 7% CO_2_ with a separate well per timepoint. At 0, 2, 8, 16, and 24 h after the start of the incubation, cells were removed from the appropriate wells and washed by centrifugation in RPMI-1640. For confocal microscopy, Hoechst 33342 was added to a final concentration of 5 μg/ml thirty minutes prior to the end of each incubation time.

Cells were resuspended in 200 μl of R10 and added to Ibidi 8 well glass bottomed plates. Images were acquired using a Leica TCs-SP8 laser scanning confocal microscope on a DMi8 inverted microscope stand with a HC PL APO CS2 63x /1.30 glycerol immersion objective zoom 2.25 and Leica LAS-X acquisition software at 37°C. Excitation lengths of 405 nm and 488 nm were used for Hoechst 33342 and the Alexa Fluor 488 conjugates respectively.

For flow cytometry, cells were resuspended in 100 μl of RPMI-1640 in flow tubes and analysed at 10 μl/min on a Cytoflex flow cytometer (Beckman Coulter) equipped with 488 nm 50 mW laser. Approximately 10,000 events were recorded per sample. Alexa Fluor 488 data was collected with a 525/40 nm bandpass filter with height (FITC-H), width (FITC-W) and area parameters recorded. Data was recorded and analysed using CytExpert software (Version 2.1.0.92, Beckman Coulter Life Sciences, Indianapolis, IN, USA).

### Colocalisation Studies

Daudi and HSB-2 cells were incubated with 1 × 10^−6^ M SAP-AF for 24 h in R10 at 37°C, 7% CO_2_. Cells were washed in RPMI-1640 and fixed in 4% PFA before being set in 2% low melting point agarose. Cells were permeabilised with 0.2% Triton X-100 and incubated with either chicken anti EEA1 or mouse anti LAMP-1, washed, incubated with goat anti-chicken Alexa Fluor 555 or goat anti-mouse Alexa Fluor 568, washed and mounted. Images were acquired using a Leica TCs-SP8 laser scanning confocal microscope on a DMi8 inverted microscope stand with a HC PL APO CS2 63x /1.30 glycerol immersion objective zoom 2.25 and Leica LAS-X acquisition software at 37°C. Excitation lengths of 405 nm and 488 nm were used for Hoechst 33342 and the Alexa Fluor 488 conjugates respectively.

### Live Cell Imaging

Daudi and HSB-2 cells were incubated with 1 × 10^−6^ M SAP-AF or 5 × 10^−6^ M OKSAP-AF in R10 at 37°C, 7% CO_2_ for 24 h. Hoechst 33342 was added to a final concentration of 5 μg/ml thirty minutes prior to the end of each incubation time. Cells were washed, resuspended in 200 μl of R10 with or without SA at 1 μg/ml or 5 μg/ml, added to Ibidi 8 well glass bottomed plates and incubated at 37°C, 7% CO_2_. Images were acquired using a Leica TCs-SP8 laser scanning confocal microscope at 37°C after 0, 8, 16 and 24 h of incubation. The gain on the FITC channel was set at t = 0 h and not modified for the duration of the experiment. In order to maximise the sensitivity of the microscope to detect low levels of cytosolic fluorescence, the gain was set so that the fluorescent intensity of endosomal compartments at this time point were saturating. For inhibitor experiments, chloroquine was added to Daudi cells prior to the addition of SA at a final concentration of 100 μM.

### Flow Cytometry

Daudi cells were incubated with 1 × 10^−6^ M SAP-AF or 5 × 10^−9^ M OKSAP-AF in R10 at 37°C, 7% CO_2_ for 24 h. This was repeated with HSB-2 cells with 1 × 10^−6^ M SAP-AF or 5 × 10^−9^ M OKSAP-AF. Cells were washed and resuspended in R10 before being plated in 96 well plates at 1.25 × 10^5^ cells per well with or without SA at 5 μg/ml, 1 μg/ml or 0.1 μg/ml in a final volume of 250 μl. Plates were incubated at 37°C, 7% CO_2_ and cells removed from appropriate wells at 0 and 24 h after the addition of SA. Cells were washed, resuspended in 100 μl of RPMI-1640 in flow tubes and the FITC-H and FITC-W of 10,000 events were measured on a Cytoflex Flow Cytometer (Beckman Coulter) using CytExpert software (Version 2.1.0.92, Beckman Coulter Life Sciences, Indianapolis, IN, USA). For inhibitor experiments, chloroquine was added to Daudi cells prior to the addition of SA at a final concentration of 100 μM.

### XTT Cytotoxicity Assay

Quadruplicate cultures of Daudi cells (5 × 10^4^ cells per well) were seeded into a 96 well plate and treated with increasing concentrations of saporin in the presence or absence of 1 μg/ml or 5 μg/ml of SA. Cells were also cultured with and without 100 μM of chloroquine. Plates were incubated for 48 h at 37°C, 7% CO_2_. Cell viability was determined by a modified XTT assay as first described by Scudiero et al. [27]. Plates were read on a BMG Fluostar plate reader using a spectral scan from 300 to 650 nm. Results were expressed as a percentage of control cells cultured in the medium or SA alone and the 50% inhibitory concentration (EC_50_) was determined from the intercept with the 50% level on the Y axis of the dose-response curve. The fold increase was calculated by dividing the EC_50_ value for IT without SA by the EC_50_ value with SA. All experiments were repeated three times.

### Statistical Analysis

The Student’s t test was used to determine *p* values for flow cytometry data comparing median FITC-W values from three independent experiments.

## Results

### SAP-AF and OKSAP-AF Accumulate in the Endolysosomal Compartment

**Fig. 2:**
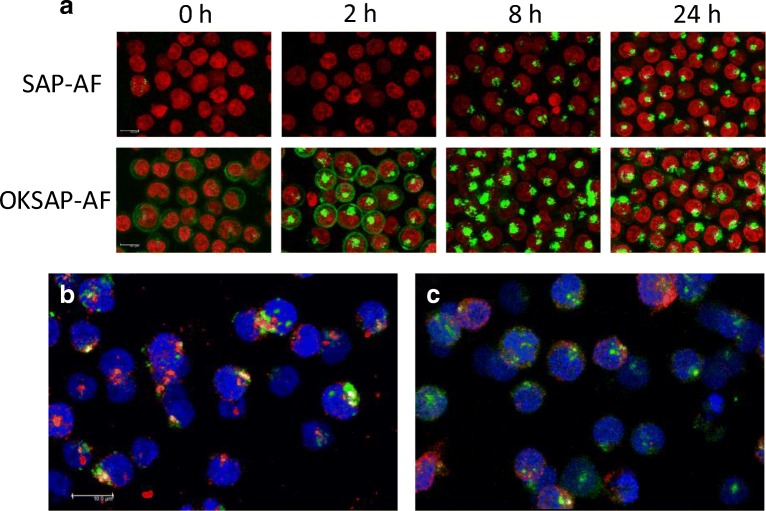
The uptake of SAP-AF and OKSAP-AF into Daudi cells. (a) Daudi cells were incubated with SAP-AF or OKSAP-AF and live cell confocal images taken after 0, 2, 8 and 24 h. The nucleus (red) was stained with Hoechst 33342. Co-localisation studies were performed between SAP-AF (green) and (b) the lysosomal marker LAMP-1 (red) or (c) the early endosomal marker EEA-1. Sites of co-localisation appear in yellow. The nucleus (blue) was stained with Hoechst 33342. Images presented are maximum projections of 21 × 1 μm Z-stacks. Scale bar represents 10 μm

In order to image the endolysosomal escape of the RIP saporin and the saporin based IT OKT10-SAP the fluorescent conjugates SAP-AF and OKSAP-AF were constructed. Both conjugates were incubated separately with Daudi and HSB-2 cells and confocal imaging was performed at time intervals to track the uptake of the conjugate into the cell. Endocytosis of SAP-AF was observed as punctate fluorescence in HSB-2 cells after two hours (Fig. S2) and in Daudi cells after eight hours (Fig. 2A). In both of these cell lines SAP-AF was not detected on the plasma membrane surface. OKSAP-AF was clearly observed bound to the plasma membrane of Daudi cells and to a lesser extent of HSB-2 cells immediately after initial exposure, internalised OKSAP-AF was observed in both cell lines after two hours (Figs. 2A and S1). Increasing length of exposure resulted in a reduction in surface fluorescence and increased intracellular punctate fluorescence. After 24 h both SAP-AF and OKSAP-AF accumulated in discrete vesicular compartments. In Daudi cells these intracellular compartments were tightly packed in a single peri-nuclear region but in HSB-2 cells intracellular compartments were more widely distributed throughout the cytosol. Escape of the IT or saporin into the cytosol was observed in only a small number of cells during this time.

Such intracellular compartments have previously been shown to be late endosomes and lysosomes [11,28]. Using confocal microscopy of SAP-AF loaded Daudi or HSB-2 cells we were able to show that in both Daudi and HSB-2 cells the toxin co-localised with the lysosome specific protein LAMP-1 and to a much lesser extent with the early endosomal marker EEA-1 (Figs. 2B + C and S1). These data indicate that after 24 h of uptake SAP-AF accumulates within the late endosome/lysosomal compartment in both cell lines.

**Fig. 3:**
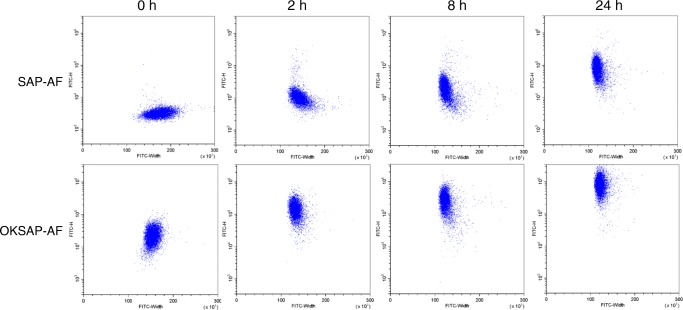
Pulse shape analysis of the uptake of SAP-AF and OKSAP-AF into Daudi cells. Daudi cells were incubated with SAP-AF or OKSAP-AF and analysed by flow cytometry after 0,2,8 and 24 h. Dot plots showing FITC-H against FITC-W parameters are presented here demonstrating the reduction in FITC-W and increase in FITC-H over time. Each displayed dot plot represents approximately 10,000 events

We next investigated whether pulse shape analysis could be used to observe the uptake of SAP-AF or OKSAP-AF into the endolysosomal compartment. Flow cytometric measurement of Daudi cells exposed to SAP-AF or OKSAP-AF for varying lengths of time showed a gradual, time dependent reduction in FITC-W, accompanied by a concomitant increase in FITC-H as illustrated in Fig. 3. This change would correspond with the uptake of the toxin from its initial, diffuse, surface bound location, as recorded at the zero-hour time point in fig. 3, into endosomal compartments via an undefined endocytic process. Trafficking of the IT or native toxin down the endosomal pathway into compartments progressively further from the plasma membrane results in a smaller area of fluorescent signal, resulting in the reduction in FITC-W that is observed at later time points. It also leads to the increased concentration of the fluorescent label and thus an increase in fluorescent intensity resulting in a higher FITC-H. Similar results were obtained in HSB-2 cells (Fig. S3).

After 24 h median FITC-H levels were significantly higher in SAP-AF and OKSAP-AF positive cells than in negative control cells and gating demonstrated that almost all the cells recorded had internalised SAP-AF. The range of FITC-W for SAP-AF and OKSAP-AF positive Daudi cells was narrow, suggesting that the intracellular distribution of SAP-AF was similar in the majority of cells corresponding to the peri-nuclear grouping observed on confocal microscopy. In HSB-2 cells a wider range of FITC-W was observed, this is likely due to the more diffuse position of the endolysosomal compartment in these cells.

### Confocal Analysis of Endolysosomal Escape

**Fig. 4:**
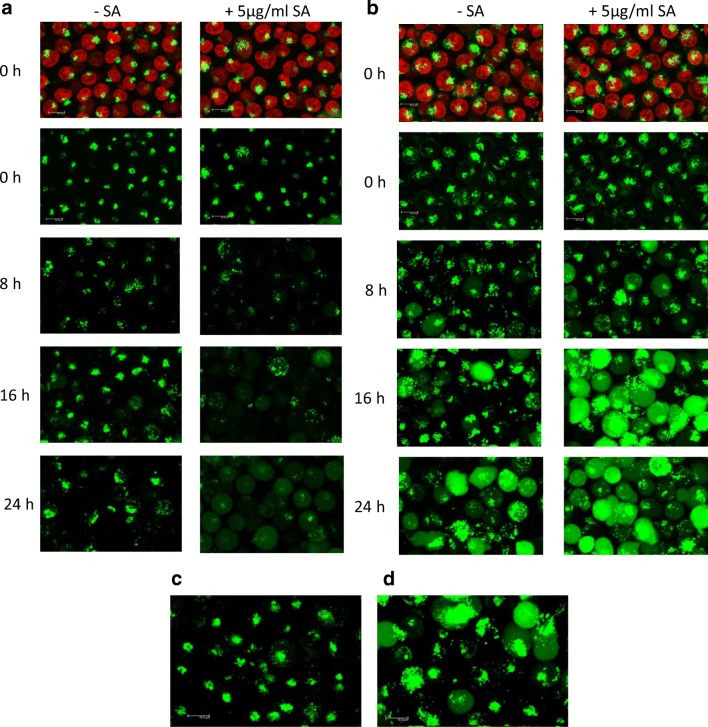
Endolysosomal escape of SAP-AF and OKSAP-AF into the cytosol. Daudi cells incubated with (a) SAP-AF or (b) OKSAP-AF for 24 h were treated with 5 μg/ml of SA and live cell confocal images taken after 0, 8, 16 and 24 h. Untreated cells are shown for comparison. Endolysosomal escape is seen as a change from punctate vesicular fluorescence to a diffuse staining pattern throughout the cytoplasm. Images for the initial timepoint at zero hours are shown with and without Hoechst 33342 nuclear stain (red). The lack of an observable increase in endolysosomal escape of (c) SAP-AF or (d) OKSAP-AF, compared to untreated control cells, in Daudi cells treated with 1 μg/ml of SA for 24 h is shown. The increased brightness of cells incubated with OKSAP-AF and treated with SA at the 16 and 24 h timepoints is due to the way the fluorescence gain was set to maximise assay sensitivity. See methods section for details. Images presented are maximum projections of 21 × 1 μm Z-stacks. Scale bar represents 10 μm

To investigate the effect of SA on the endolysosomal escape of saporin and IT, Daudi cells loaded with SAP-AF or OKSAP-AF were observed and recorded by confocal microscopy at time intervals after the addition of a range of concentrations of SA. Untreated control cells were used as comparator. Endolysosomal escape of both SAP-AF and OKSAP-AF was observed in a small proportion of cells eight hours after the addition of 5 μg/ml of SA (Fig. 4A and B). This could be seen as a reduction in endolysosomal fluorescence and the diffusion of the fluorescently labelled toxin throughout the cytosol. At later time points the proportion of cells showing cytosolic fluorescence increased, with almost all cells showing escape by 24 h (Fig. 4A and B). The intensity of cytosolic fluorescence increased over time demonstrating the continuing endolysosomal escape of SAP-AF and OKSAP-AF. Similar results were obtained for both SAP-AF and OKSAP-AF in HSB-2 cells. (Figure S4).

In comparison, in untreated control cells, endolysosomal escape was only observed in a small number of cells imaged, even after 24 h. These results demonstrate the effect of this moderate concentration of SA as an endolysosomal escape enhancer for saporin and saporin based ITs. The concentration of 5 μg/ml of SA is partially cytotoxic in both Daudi and HSB-2 cells as measured by XTT assay after 48 h of exposure [17]. At the subtoxic but IT augmentative concentration of 1 μg/ml of SA there was no apparent increase in endolysosomal escape of either saporin or IT by confocal microscopy above that seen in untreated control cells (Fig. 4C and D).

### Evaluation of Endolysosomal Escape by Pulse Shape Analysis

**Fig. 5:**
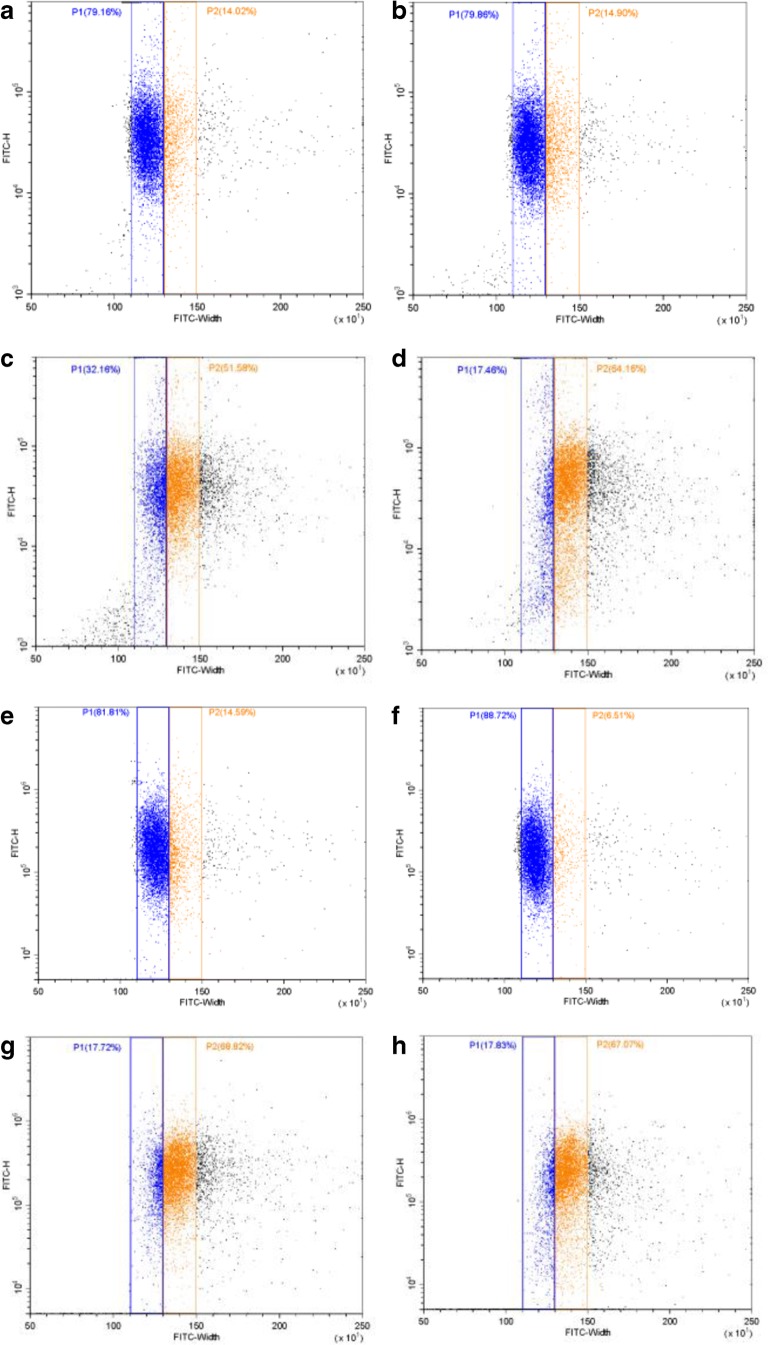

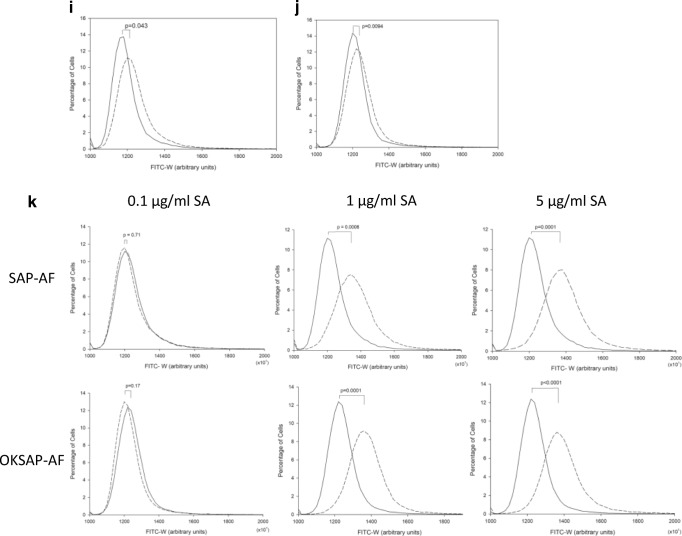
The use of pulse width analysis to investigate endolysosomal escape. Dot plots showing FITC-W against FITC-H for Daudi cells incubated with SAP-AF for 24 h before being treated with (b) 0.1 μg/ml, (c) 1 μg/ml or (d) 5 μg/ml of SA or (a) mock treated with R10 for a further 24 h. Experiments were also performed with Daudi cells incubated with OKSAP-AF and treated with (e) R10 or (f) 0.1 μg/ml, (g) 1 μg/ml or (h) 5 μg/ml of SA for 24 h before cells were analysed by flow cytometry. Each displayed dot plot represents approximately 10,000 events. Pulse width histograms showing the change in FITC-W for control Daudi cells preincubated with SAP-AF (i) or OKSAP-AF (j) for 24 h and then dummy treated with R10. The distribution immediately after incubation is shown with a solid line whilst the dashed line shows the distribution a further 24 h later. *P* values for the difference in median FITC-W between t = 0 h and t = 24 h are shown on each chart (*n* = 3 independent experiments). (k) Daudi cells preincubated with SAP-AF or OKSAP-AF were treated with 0.1 μg/ml, 1 μg/ml or 5 μg/ml of SA for a further 24 h. Cells treated with SA are marked with a dashed line, each chart also shows the histogram for untreated cells at the same time point, shown with a solid line. P values for the difference in median FITC-W between treated and untreated cells are shown on each chart (n = 3 independent experiments)

We aimed to explore the use of pulse width analysis to quantify the endolysosomal escape that we had observed by confocal microscopy. To confirm that analysis of FITC-W was able to discriminate between SAP-AF or OKSAP-AF located in the endolysosomal compartment from that located in the cytosol, SAP-AF and OKSAP-AF positive Daudi and HSB-2 cells were treated with a range of concentrations of SA (0.1–5 μg/ml) and analysed by flow cytometry at 0 and 24 h after the addition of SA (Fig. 5 and S4).

Control cells, mock treated with R10, showed a significant increase in median FITC-W over 24 h (Fig. 5I + J). This result agrees with the confocal imaging that showed endolysosomal escape in a small number of control cells in the absence of saponin (Fig. 4). Cells treated with the various concentrations of SA were therefore compared to control cells at 24 h post treatment. A significant increase in median FITC-W was seen in SAP-AF and OKSAP-AF loaded Daudi cells treated with SA at 5 μg/ml when compared to control cells. This confirmed that the increased levels of endolysosomal escape observed by confocal microscopy could be measured by pulse shape analysis and that they corresponded with an increase in FITC-W as predicted.

A smaller but still significant increase in FITC-W was also seen in Daudi cells treated with 1 μg/ml of SA with both SAP-AF and OKSAP-AF (Fig. 5). This concentration is sub-toxic in both cell lines but is capable of augmenting the cytotoxicity of both saporin and OKT10-SAP. Such an increase in endolysosomal escape was not seen by confocal imaging. We propose this as evidence that this concentration of SA induces a slower rate of escape of the labelled toxin from the endolysosomal compartment resulting in lower cytosolic concentrations that are below the level of sensitivity of confocal microscopy. In Daudi cells treated with 0.1 μg/ml SA, a concentration that is non-augmentative for IT or saporin cytotoxicity, no increase in FITC-W was observed indicating that there was no increase in endolysosomal escape over that seen in control cells (Fig. 5).

In the HSB-2 cell-line there was a significant increase in FITC-W in cells loaded with SAP-AF or OKSAP-AF and then treated with 5 μg/ml of SA for 24 h (Fig. S5). However, in cells treated with 1 μg/ml of SA no significant increase was seen at 24 h. However, increasing the length of exposure of HSB-2 cells to 1 μg/ml SA to 48 h resulted in a significant increase in FITC-W with both saporin and the IT compared with untreated controls (Fig. S5). No increase in FITC-W was observed with 0.1 μg/ml of SA even after 48 h.

### Inhibition of Endolysosomal Escape by Chloroquine

To support the conclusion that the increase in FITC-W observed in cells treated with 1 μg/ml of SA is due to an increase in the endolysosomal escape of SAP-AF that cannot be seen on confocal due to issues with sensitivity we looked to inhibit the endolysosomal escape process.

**Fig. 6:**
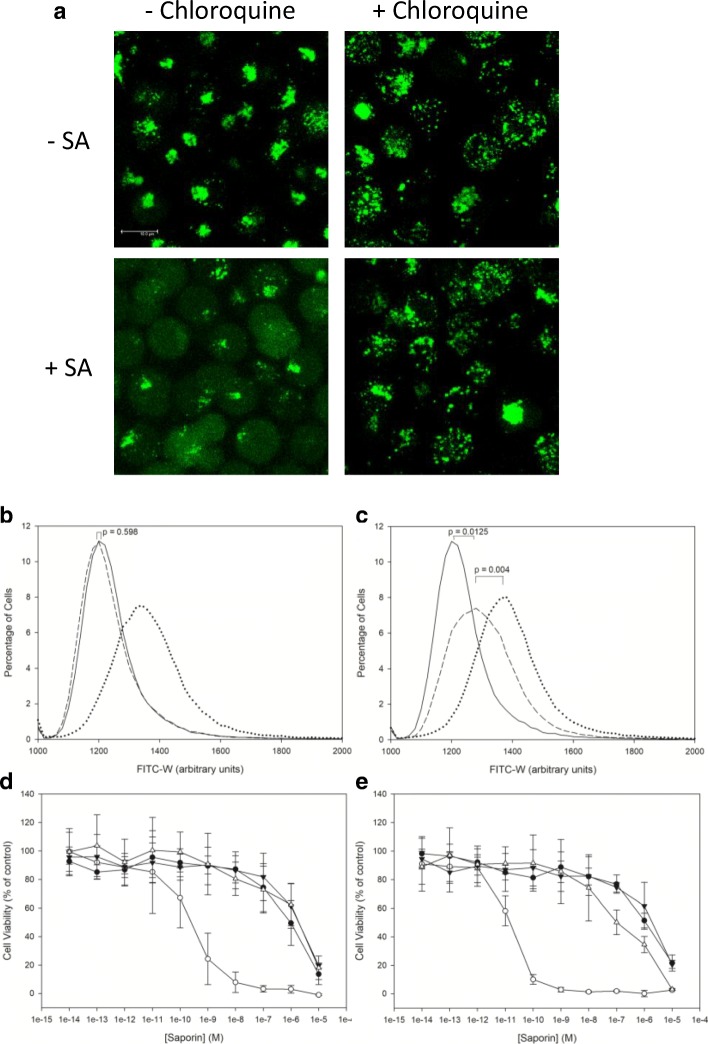
The effect of chloroquine on SA induced endolysosomal escape. (a) Live cell confocal microscopy showing Daudi cells preincubated with SAP-AF for 24 h followed by treatment with 5 μg/ml of SA in the presence or absence of chloroquine for a further 24 h. Images presented are maximum projections of 21 × 1 μm Z-stacks. Scale bar represents 10 μm. Pulse width histograms showing data from three combined experiments demonstrating the effect of chloroquine on FITC-W in SAP-AF containing Daudi cells treated with (b) 1 μg/ml or (c) 5 μg/ml of SA for 24 h. Histograms show data for untreated cells (solid line), cells treated with SA (dotted line) and cells treated with both SA and chloroquine (dashed line). P values for the difference in median FITC-W between treatments are shown on each chart (n = 3 independent experiments). Dose response curves determined by XTT assay for saporin on Daudi lymphoma cells with 100 μM chloroquine in the absence (▼) and presence (▽) of (d) 1 μg/ml or (e) 5 μg/ml of SA, the data for saporin without chloroquine in the absence (●) and presence (○) of SA is also presented for comparison. Each datum point represents the calculated mean of three independent experiments and the error bars one standard deviation either side of this mean

The lysosomotropic drug chloroquine, has previously been shown to abrogate the augmentation of both saporin and OKT10-SAP cytotoxicity by SA [29,30] and to prevent the saponin induced endolysosomal escape of an Alexa Fluor conjugated saporin toxin [11]. Chloroquine diffuses into the acidic late endosomal and lysosomal compartments where it becomes protonated, increasing the lumenal pH [31]. Such an increase in lysosomal pH is theorised to be responsible for inhibiting the SA mediated increase in endolysosomal escape based on some evidence of a pH dependent, non-covalent association between saporin and saponins at an acidic pH [16]. To confirm that chloroquine does inhibit endolysosomal escape of SAP-AF in Daudi cells, cells were loaded with SAP-AF and then treated with 1 or 5 μg/ml of SA in the presence and absence of chloroquine. Measurement of the intracellular distribution of SAP-AF by confocal microscopy after 24 h revealed that chloroquine caused a dispersal of the SAP-AF containing endolysosomal compartment throughout the cell but prevented its escape into the cytosol (Fig. 6A).

In parallel the effect of chloroquine on the SA mediated increase in FITC-W was investigated in the same bulk population of cells. Daudi cells loaded with SAP-AF were treated with 1 μg/ml or 5 μg/ml of SA for 24 h in the presence of chloroquine before the median FITC-W of each sample was measured. Chloroquine completely abrogated the increase in median FITC-W caused by 1 μg/ml of SA and significantly reduced that caused by 5 μg/ml of SA (Fig. 6B + C). From this we concluded that chloroquine prevented the endolysosomal escape of SAP-AF in cells treated with 1 μg/ml of SA, this correlates with its effect on the augmentation of saporin cytotoxicity by this concentration of SA [30]. At the higher concentration of 5 μg/ml SA, chloroquine did not completely prevent the escape of SAP-AF into the cytosol and although this could not easily be quantified by confocal microscopy, measurement of FITC-W provided a more sensitive measurement.

To investigate the relationship between the effect of chloroquine on the SA mediated increase in FITC-W and its effect on the augmentation of saporin cytotoxicity by SA, XTT cytotoxicity assays were performed (Fig. 6D + E). The augmentative effect of SA was demonstrated to be concentration dependent in line with its effect on endolysosomal escape. A mean increase in the EC_50_ of saporin of 71,100-fold was measured with 5 μg/ml of SA compared to a 3100-fold increase with 1 μg/ml of SA. The addition of chloroquine completely abrogated the augmentation of saporin cytotoxicity by 1 μg/ml of SA but the abrogation of the augmentation observed with 5 μg/ml was only partial. These results correspond with the effect of chloroquine on FITC-W and suggest that the increase in the endolysosomal escape of saporin observed with SA is a major contributor to the augmentative effect of SA on saporin/IT cytotoxicity.

## Discussion

Fluorescent pulse shape analysis is a promising but currently under-utilised application of flow cytometry. The measurement of pulse width has been previously used to investigate nuclear enlargement [19], protein aggregation states [20] and the trafficking of fluorescently labelled proteins from the cell surface to intracellular locations [22]. We reasoned that this method could be adapted to provide a high-throughput, quantitative analysis of the endolysosomal escape of protein toxins into the cytosol. Here we have demonstrated that pulse width analysis is capable of following the endocytosis of a fluorescently labelled saporin toxin and its trafficking along the endosomal pathway to the late endosomal and lysosomal compartments. Furthermore, this methodology can discriminate between cells where the fluorescent saporin is located purely within a vesicular compartment and those where the toxin has escaped into the cytosol.

The use of pulse width analysis to investigate the endolysosomal escape of toxins and immunotoxins offers several advantages over traditional methods. By providing a direct assessment of endolysosomal escape, in contrast to methods that rely on measuring the cytotoxic effect of the escaped toxin, pulse width analysis allows for specific measurement of the efficacy of endolysosomal escape enhancers. With the capacity for flow cytometry to measure thousands of cells a minute a quantitative assessment of these enhancers is possible without the requirement for complex microscopic image analysis and cell selection bias.

Our results show that flow cytometry is able to detect the presence of SAP-AF or OKSAP-AF in the cytosol at concentrations that could not be differentiated from background levels of fluorescence by confocal microscopy. Furthermore, the increased sensitivity of flow cytometry over fluorescence microscopy enables the detection of endolysosomal escape at lower concentrations of SA. This was particularly important because of the cytotoxicity of SA at higher concentrations.

Whilst pulse shape analysis has a number of advantages, there are also several limitations that need to be considered. The use of pulse width flow cytometry should be used in conjunction with fluorescence microscopy to confirm that any changes in pulse width are genuinely due to endolysosomal escape and not the result of trafficking of the fluorescent toxin to different endosomal compartments or movement of the endolysosomal organelles within the cell.

The distribution of the endolysosomal compartment within the cell may also determine the sensitivity of pulse width analysis for detection of endolysosomal escape. This can be seen when comparing the HSB-2 and Daudi cell lines. In HSB-2 cells the endolysosomal compartment is widely distributed throughout the cell in comparison to the much more clustered pattern observed in Daudi cells. This wider spread of endolysosomes resulted in a broader range of FITC-W within the cell population which may have the consequence of reducing the sensitivity of the assay.

Macromolecules, including proteins, peptides and siRNAs, are an increasingly important source of therapeutics. The large size and resulting poor bioavailability of these molecules often requires the use of a delivery system to facilitate their endocytic uptake into the cell. The rate limiting step in this delivery process is often endolysosomal escape into the cytosol and overcoming this remains a major challenge [32]. The use of pulse shape analysis to quantify endolysosomal escape may represent a useful tool in the development of escape enhancers.

## Conclusion

A flow cytometric method for the quantification of endolysosomal escape was developed and used to investigate the augmentation of the escape of the toxin saporin and the saporin based immunotoxin OKT10-SAP in cells treated with saponins. This method was able to demonstrate that saponins increased the endolysosomal escape of both OKT10-SAP and the native toxin at subtoxic concentrations, in line with those capable of augmenting cytotoxicity. The quantifiable nature of flow cytometry data enabled more sensitive detection of increases in endolysosomal escape than confocal microscopy.

## Electronic supplementary material

ESM 1 (DOCX 79215 kb)

## Abbreviations

FITC-H: Pulse height of the signal recorded by a 525/40 nm bandpass filter
FITC-W: Pulse width of the signal recorded by a 525/40 nm bandpass filter
IT: Immunotoxin
OKSAP-AF: Alexa Fluor 488 conjugated OKT10-SAP
R10: RMPI-1640 medium supplemented with 10% FCS and 2 mM glutamine and 2 mM sodium pyruvate
SA: Saponinum Album
SAP-AF: Alexa Fluor 488 conjugated saporin
