# Supplementary material for: A Flow Cytometric Method to Quantify the Endosomal Escape of a Protein Toxin to the Cytosol of Target Cells
Source: Pharm Res. 2019 Dec 23;37(1):16. doi: 10.1007/s11095-019-2725-1 (PMC6928089; doi:10.1007/s11095-019-2725-1)
Supplement: Supplementary file 1 — (DOCX 79215 kb) [file 11095_2019_2725_MOESM1_ESM.docx]

**Figure S1**


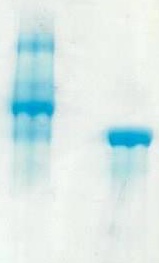

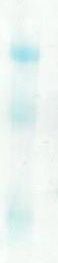


OKT10-SAP

OKT10

kDa

MW

Native Ab

1:1

1:2

1:3?

250

150

100


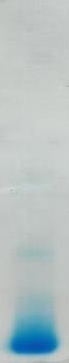

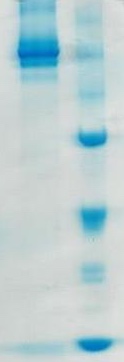


OKT10-SAP

MW

Saporin

250

150

100

75

25

50

37

kDa

A

B

**Figure S2**


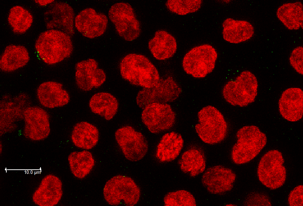

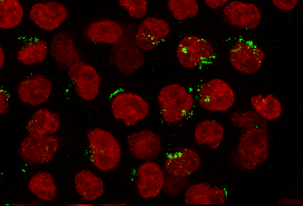

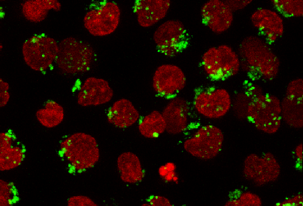

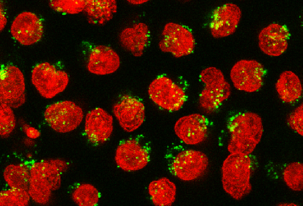


0 h

8 h

24 h

2 h

SAP-AF


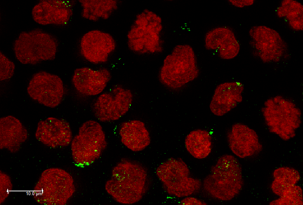

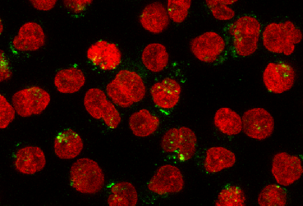

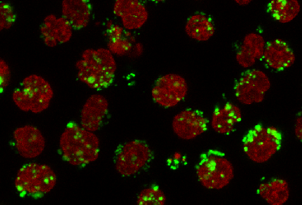

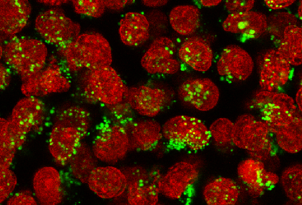


OKSAP-AF

A

**
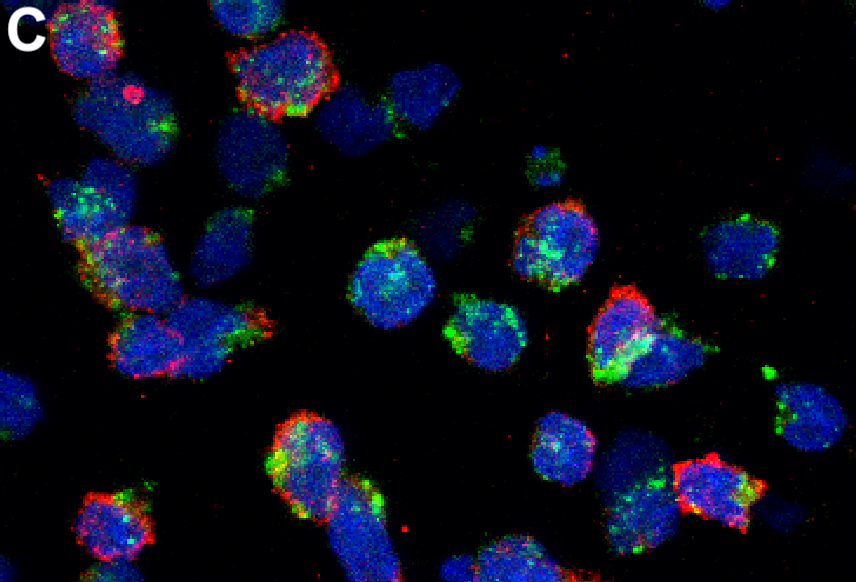

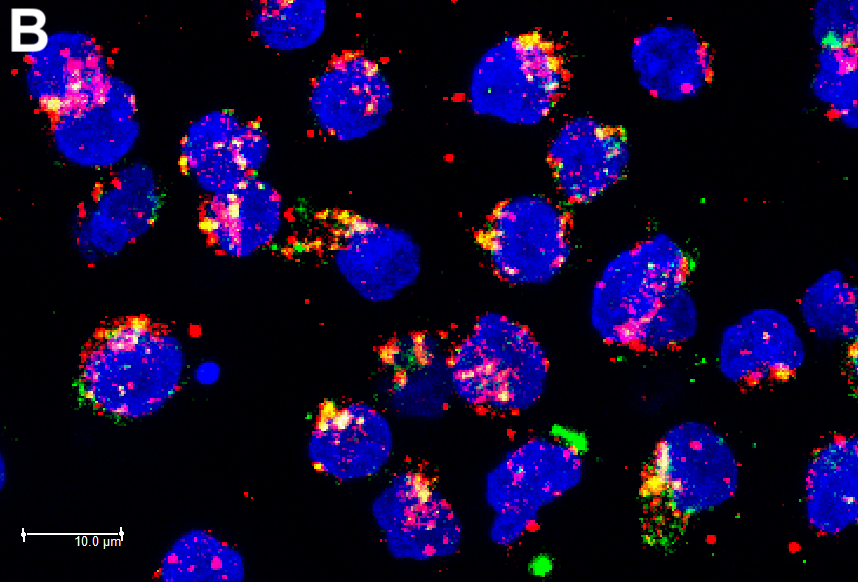
**

**Figure S3**

0 h

8 h

24 h

2 h

SAP-AF

OKSAP-AF


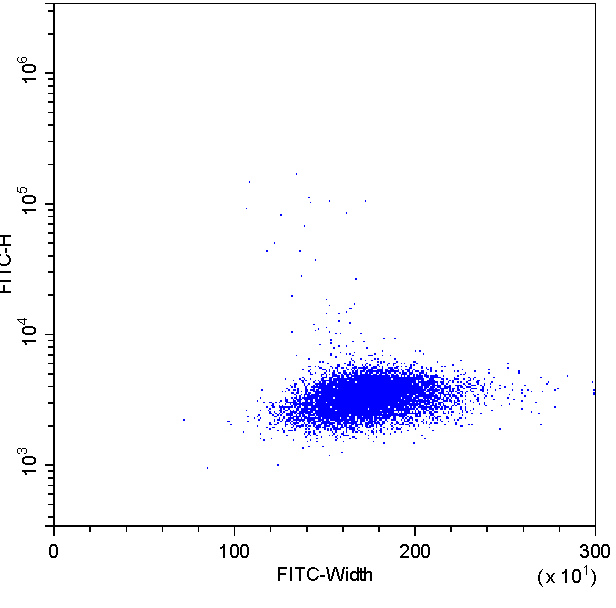

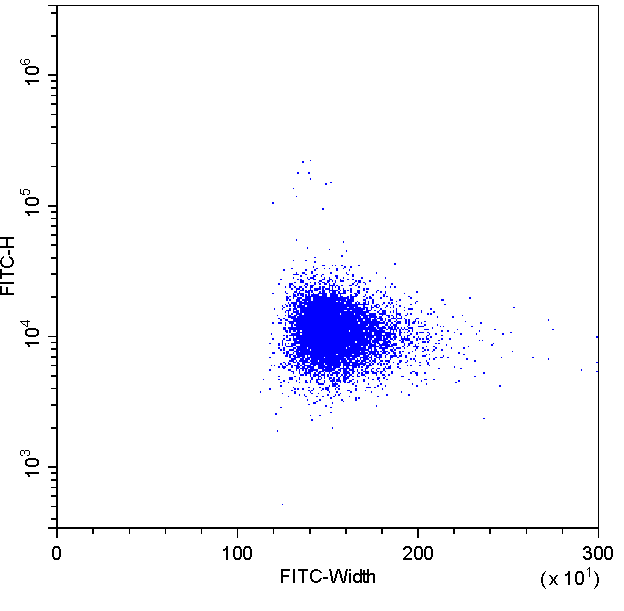

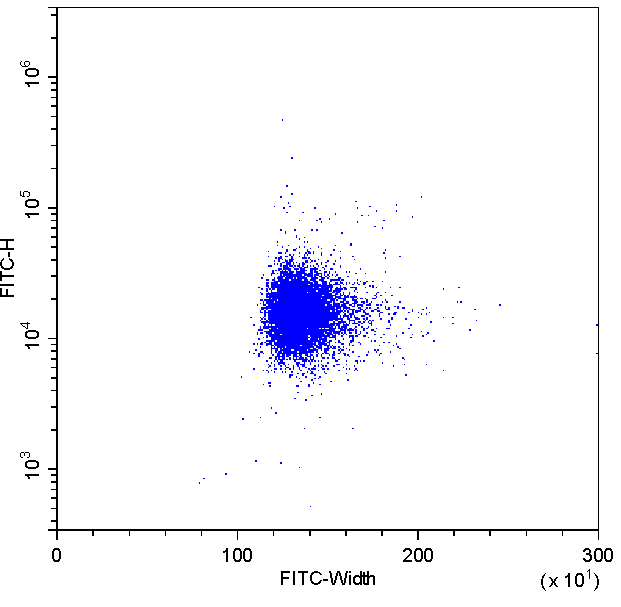

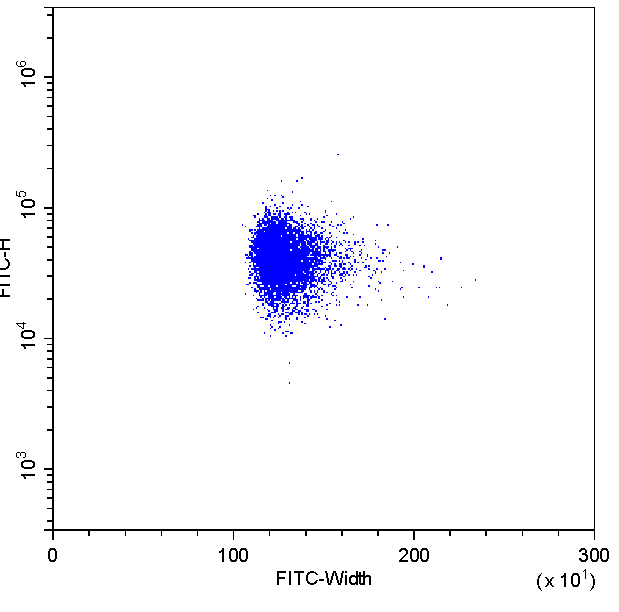

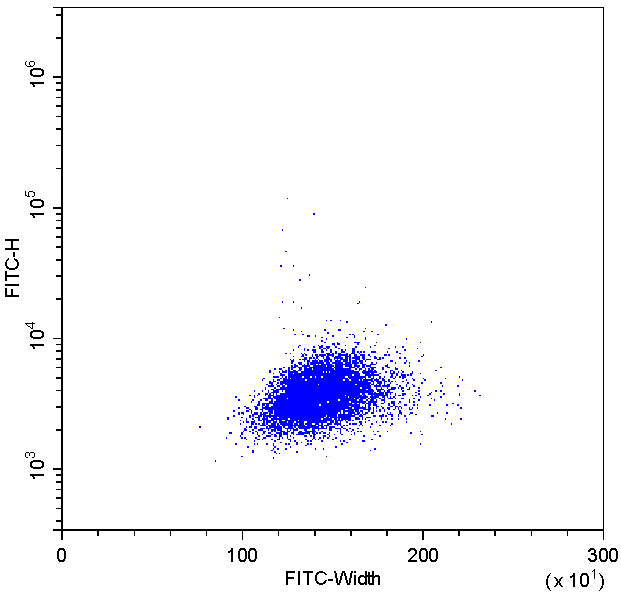

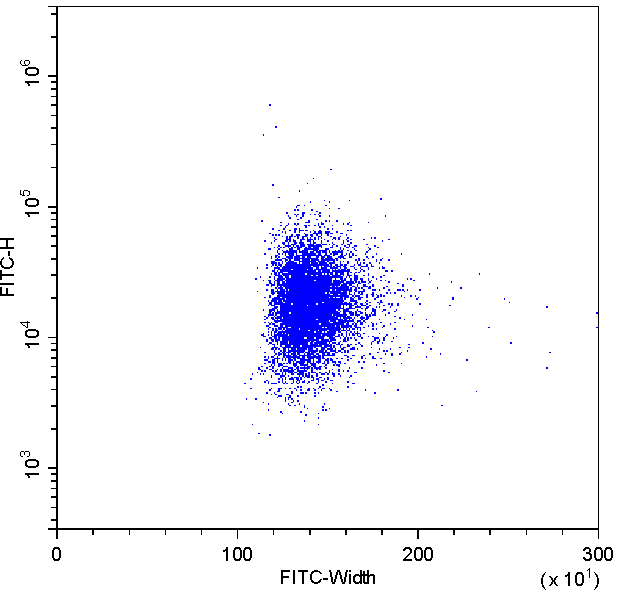

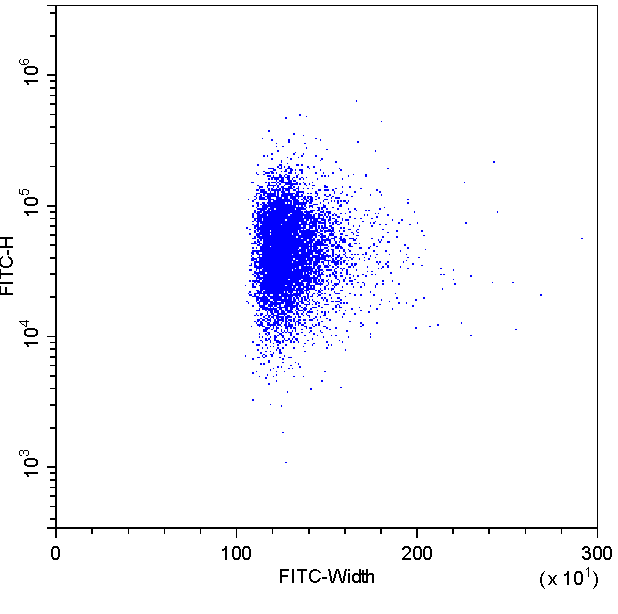

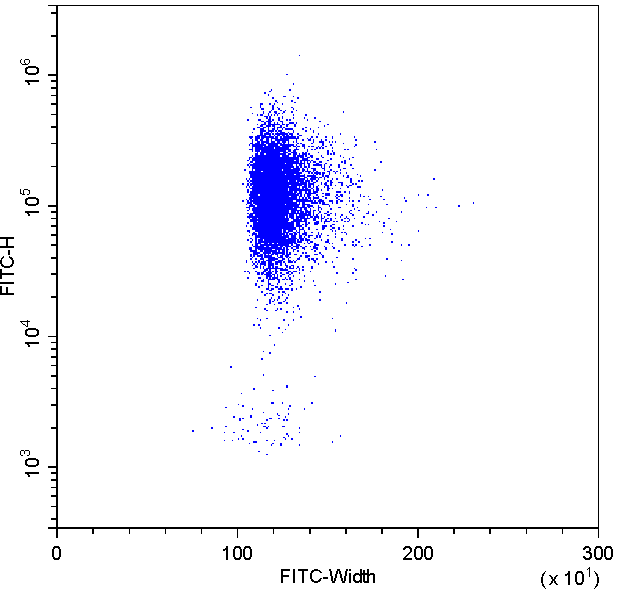


**Figure S4**


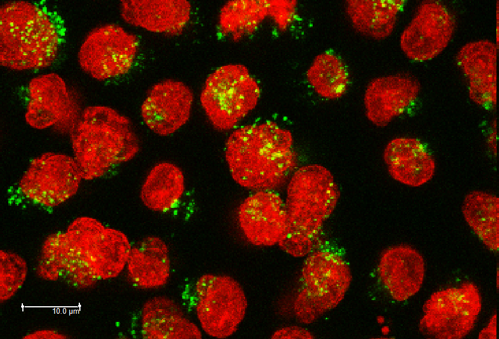

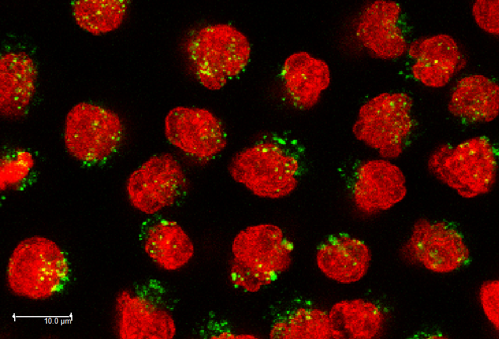


- SA

+ 5µg/ml SA

0 h


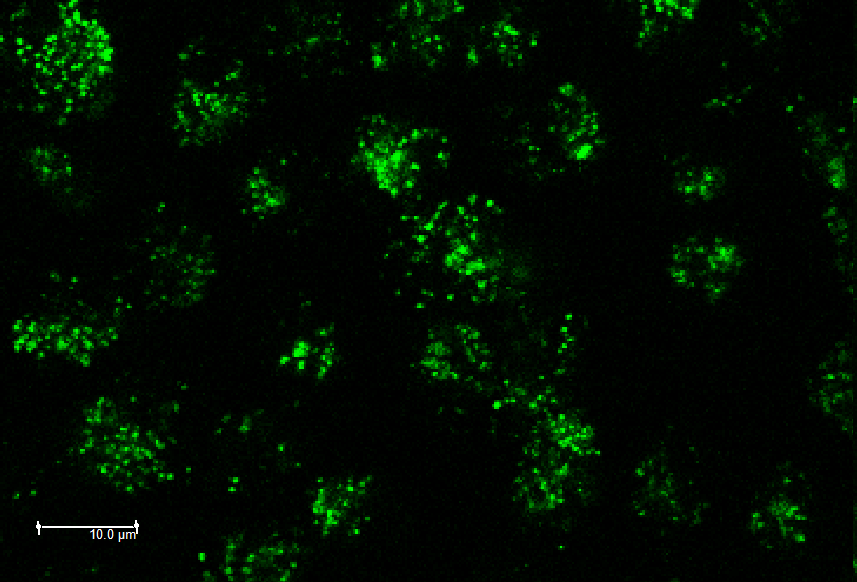

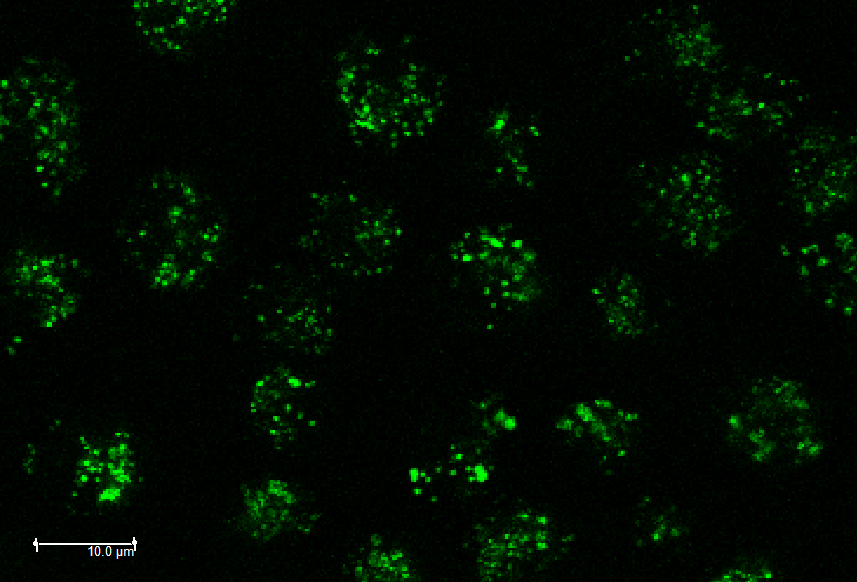

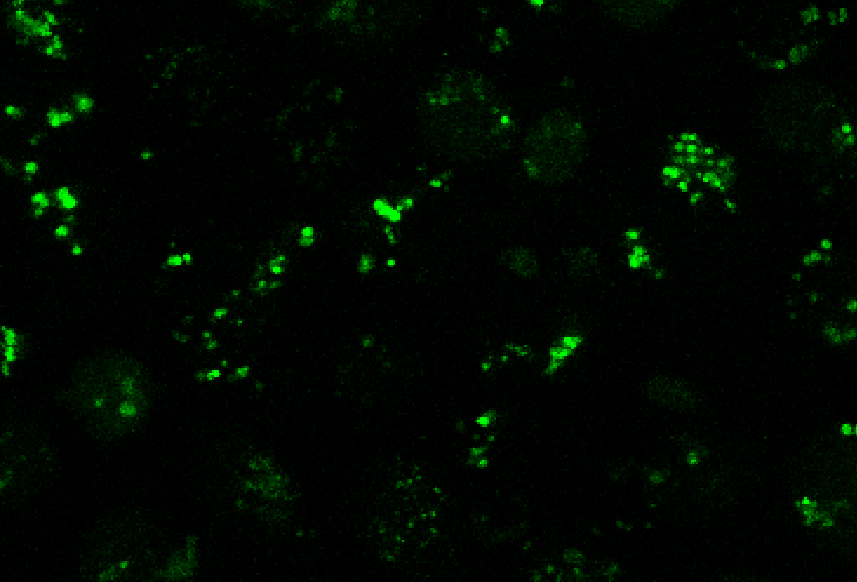

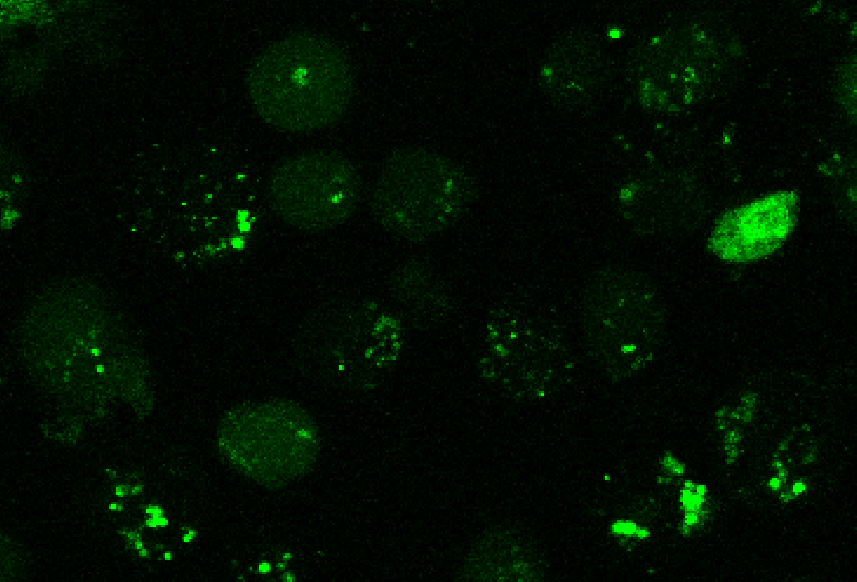

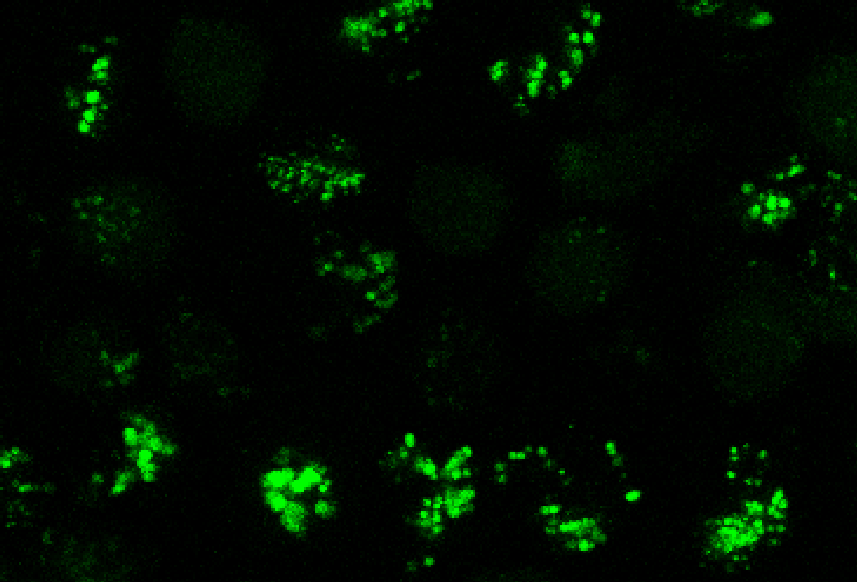

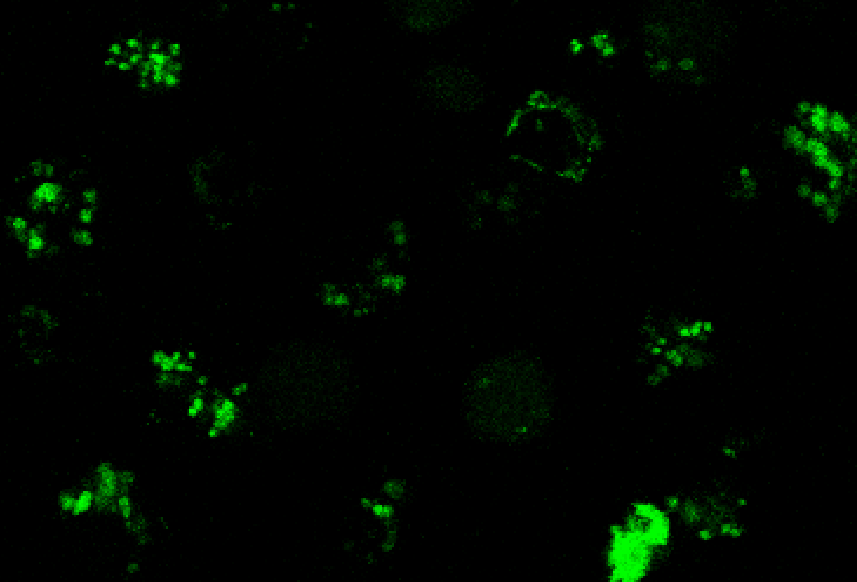

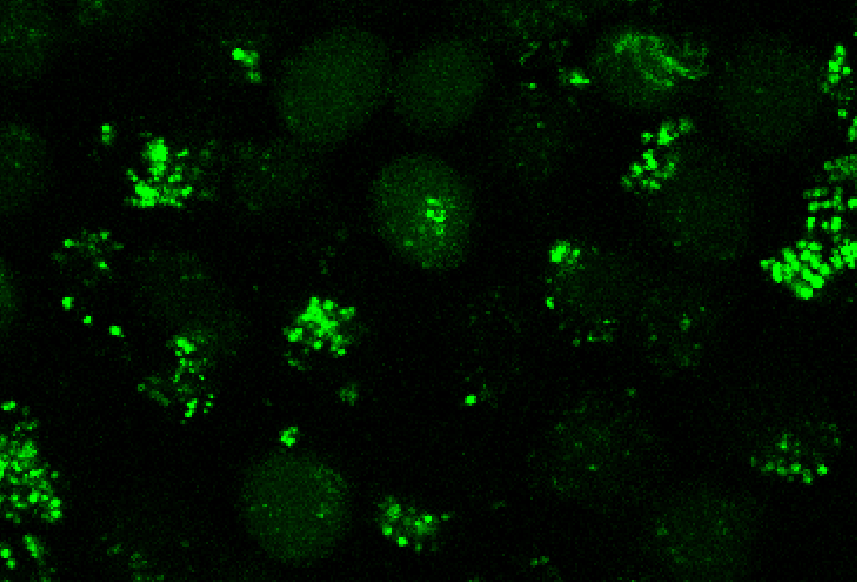

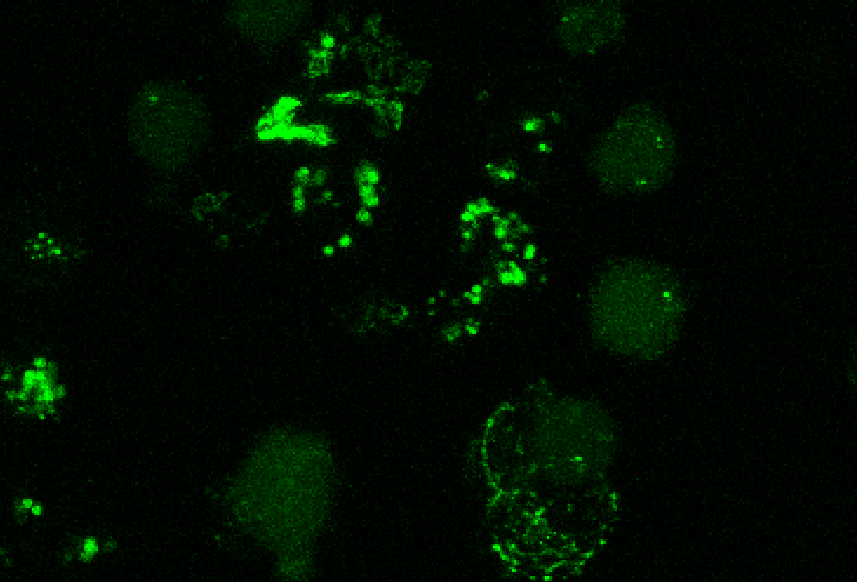


0 h

8 h

16 h

24 h

A


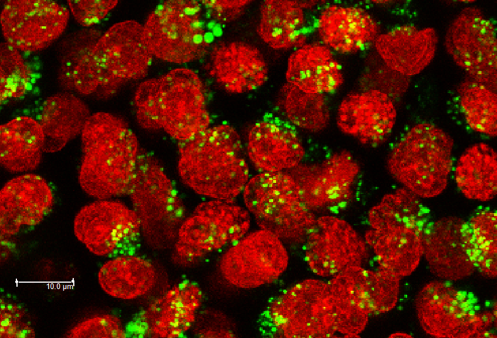

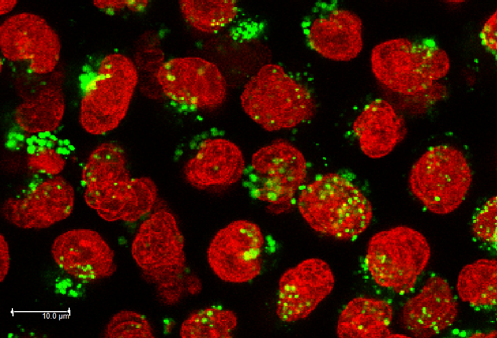


- SA

+ 5µg/ml SA

0 h

0 h

8 h

16 h

24 h

B


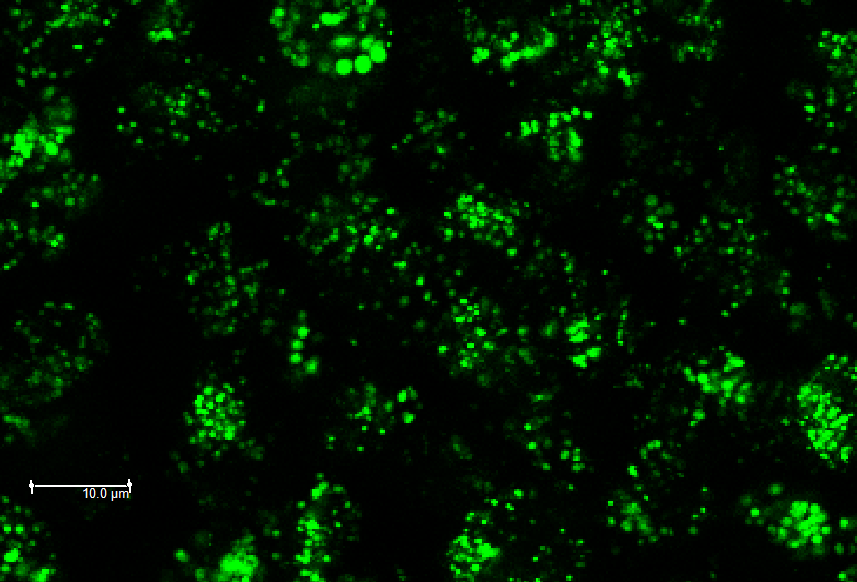

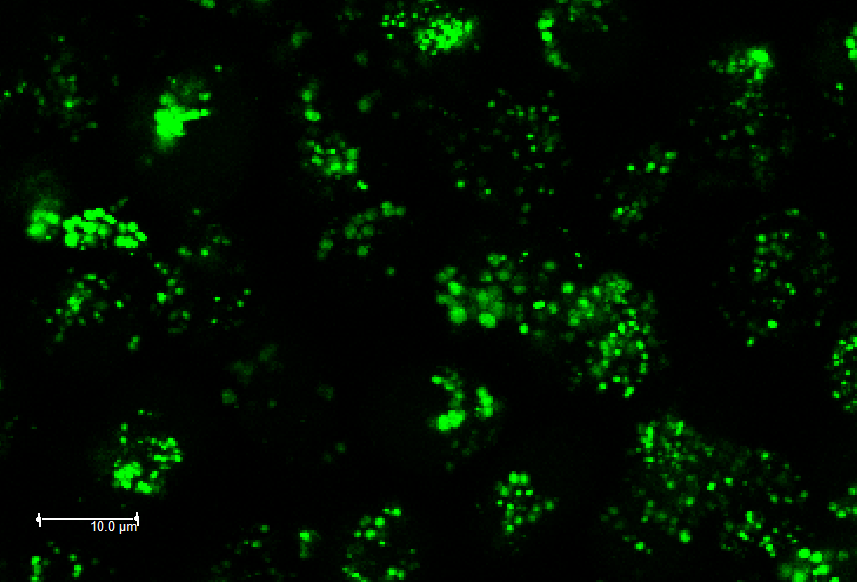

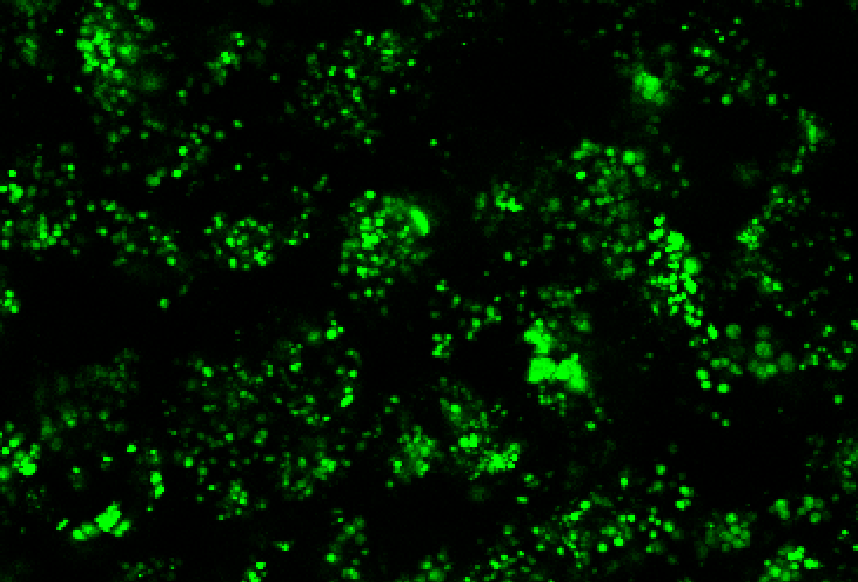

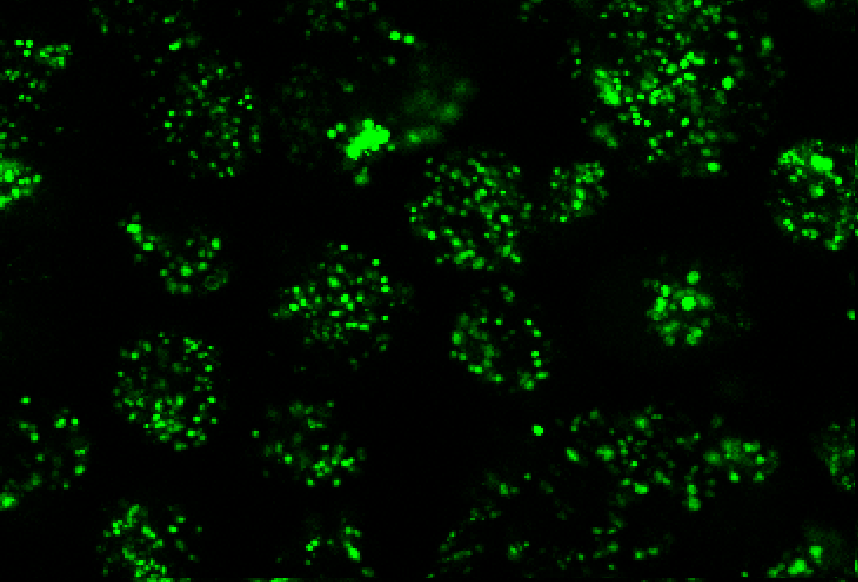

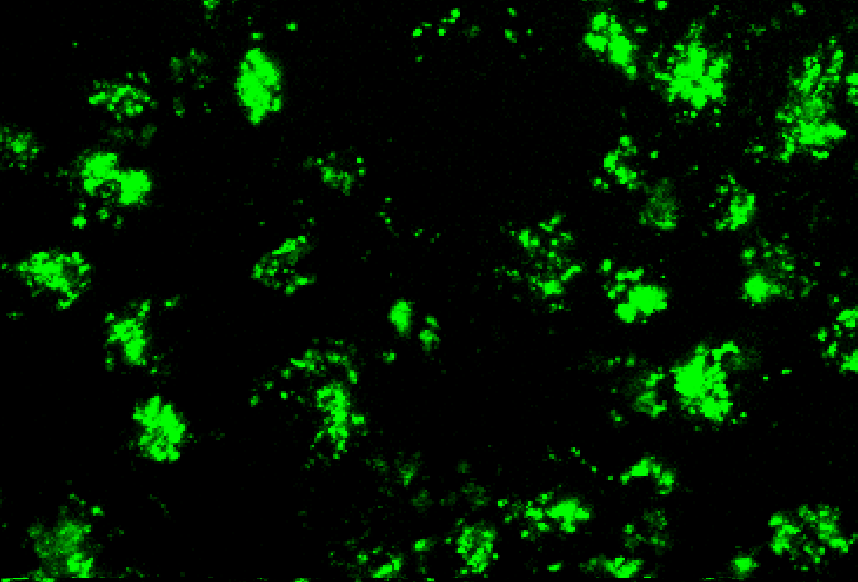

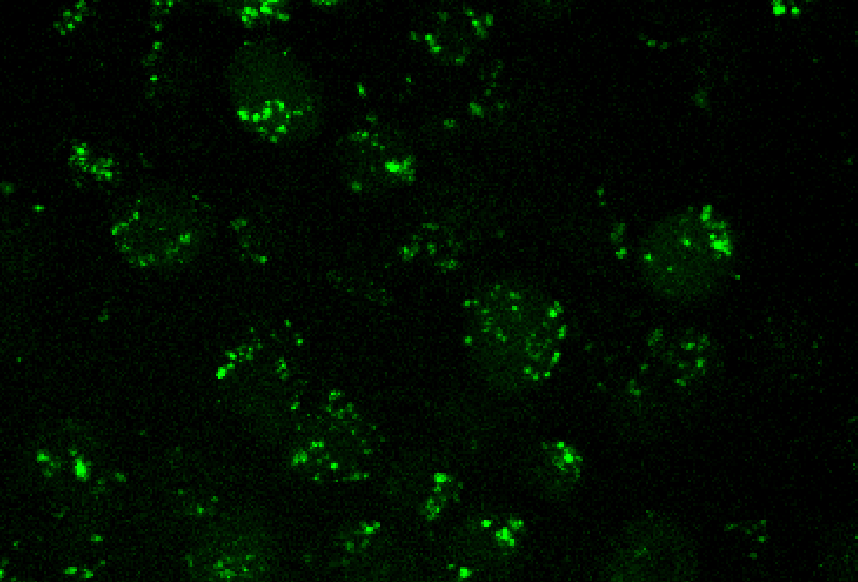

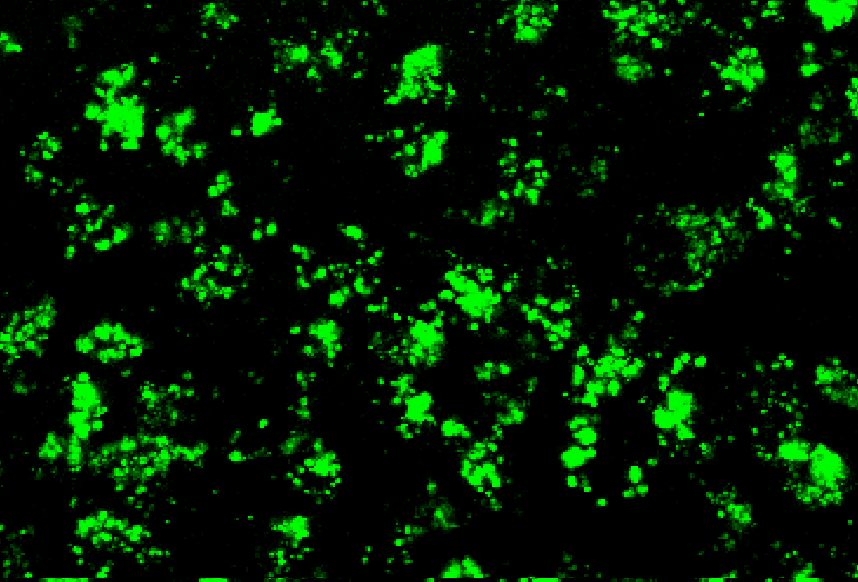

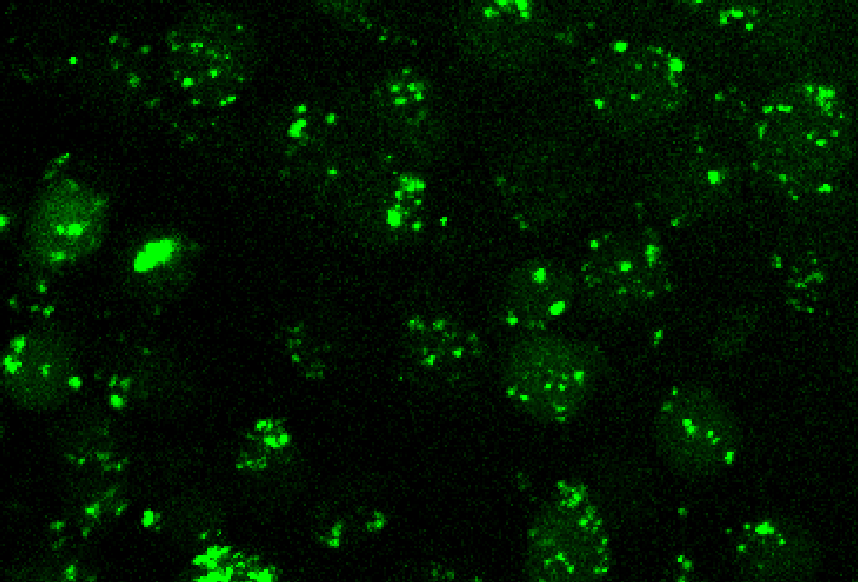


**Figure S5**


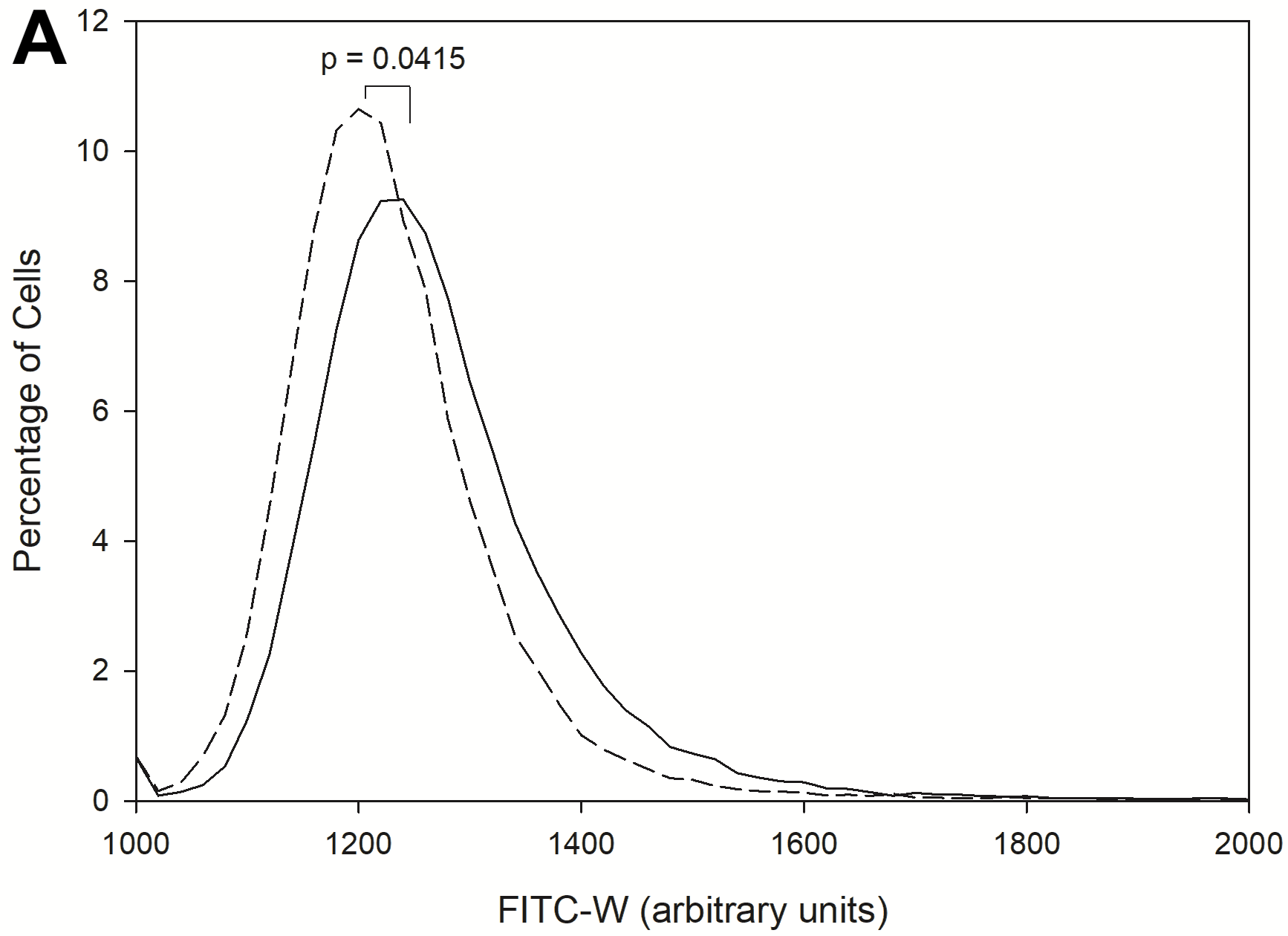

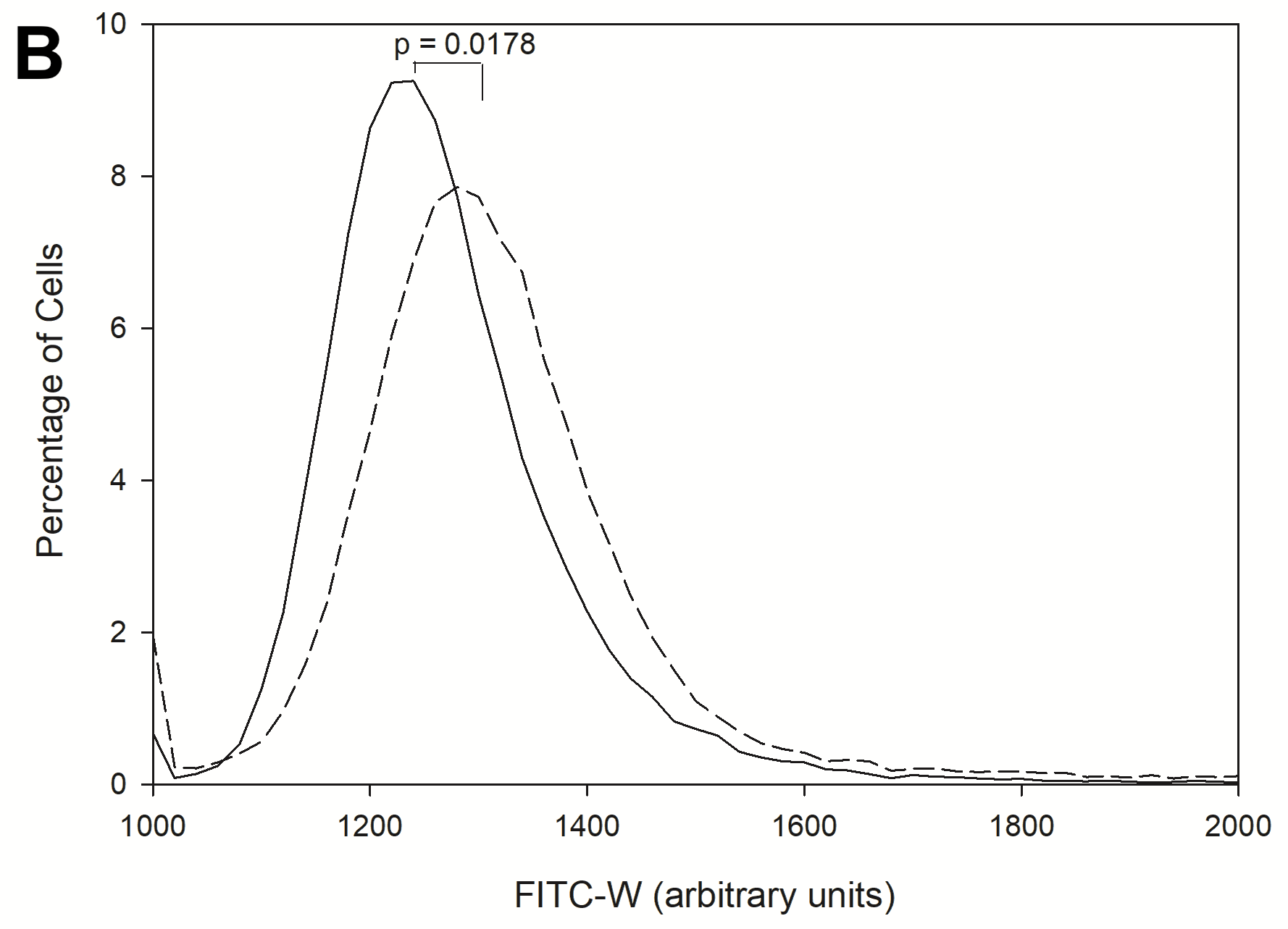

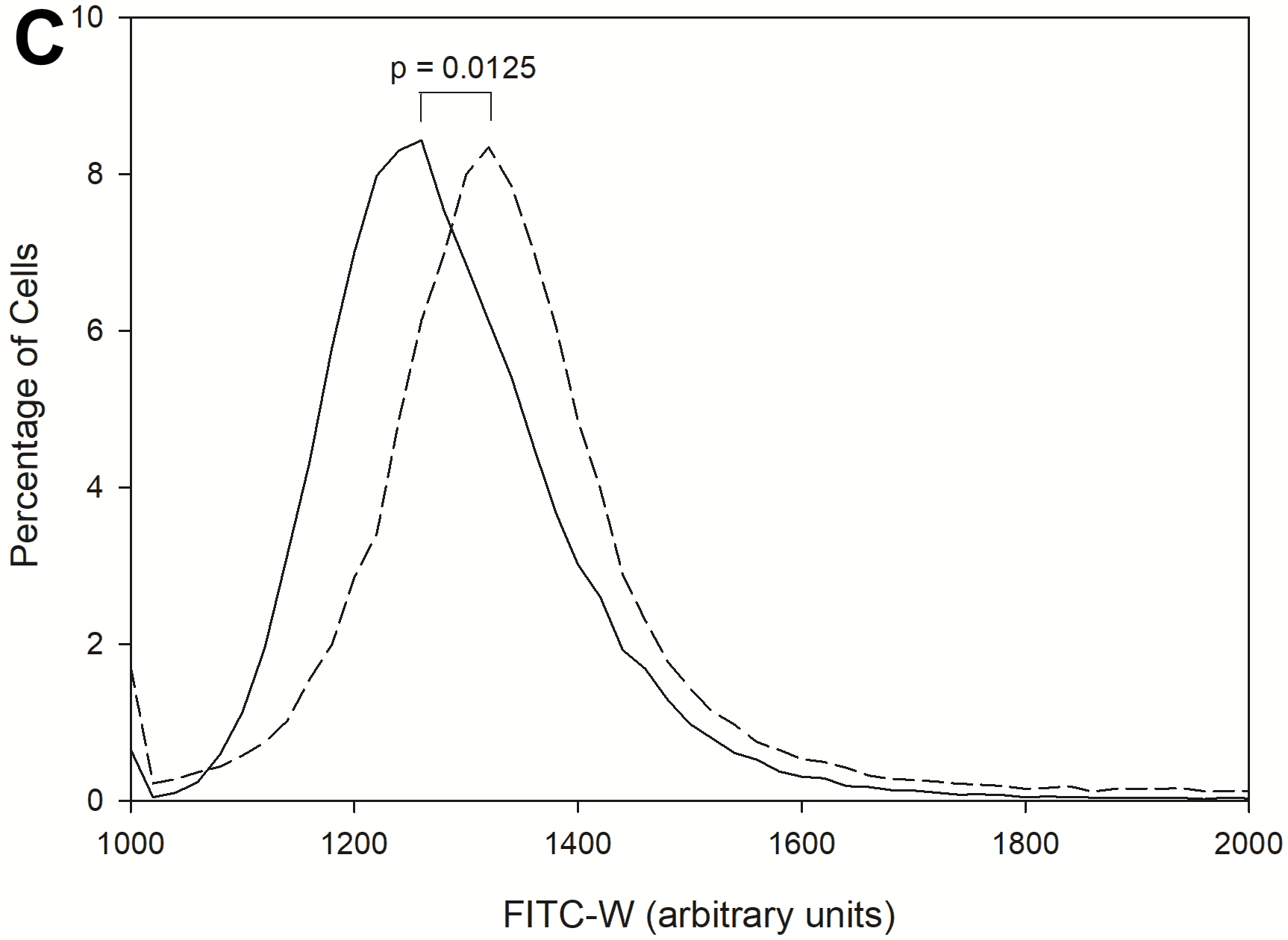

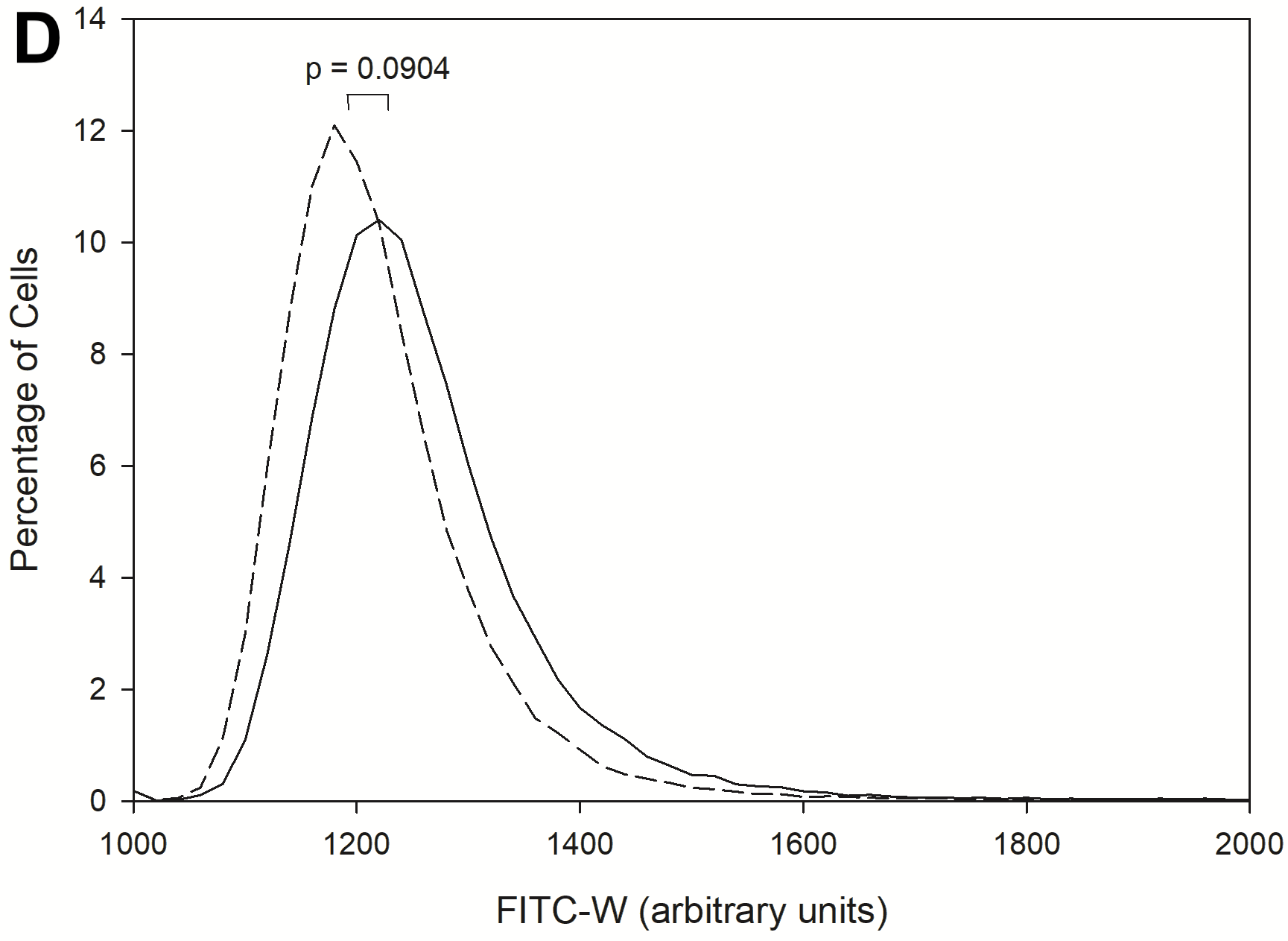

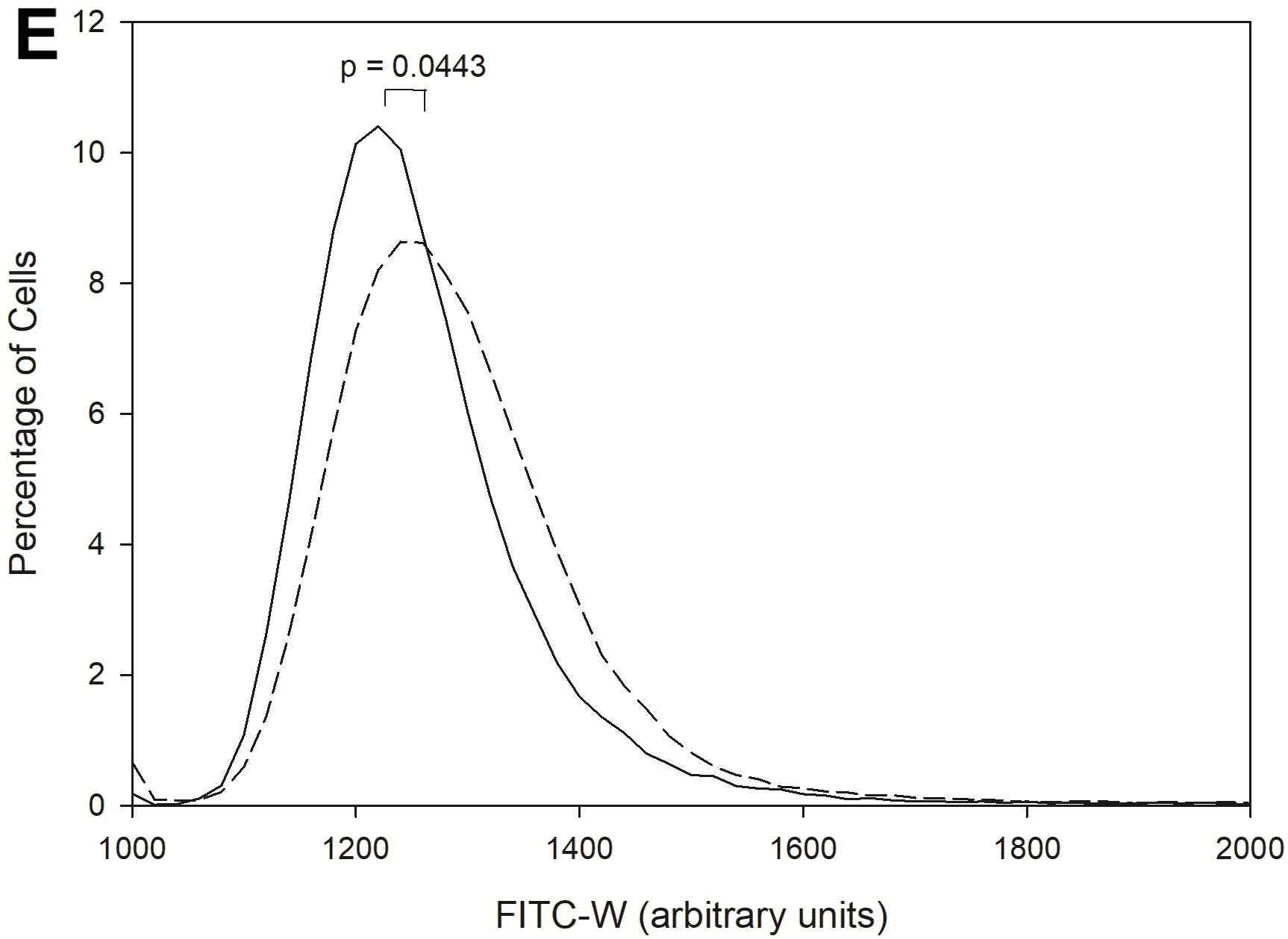

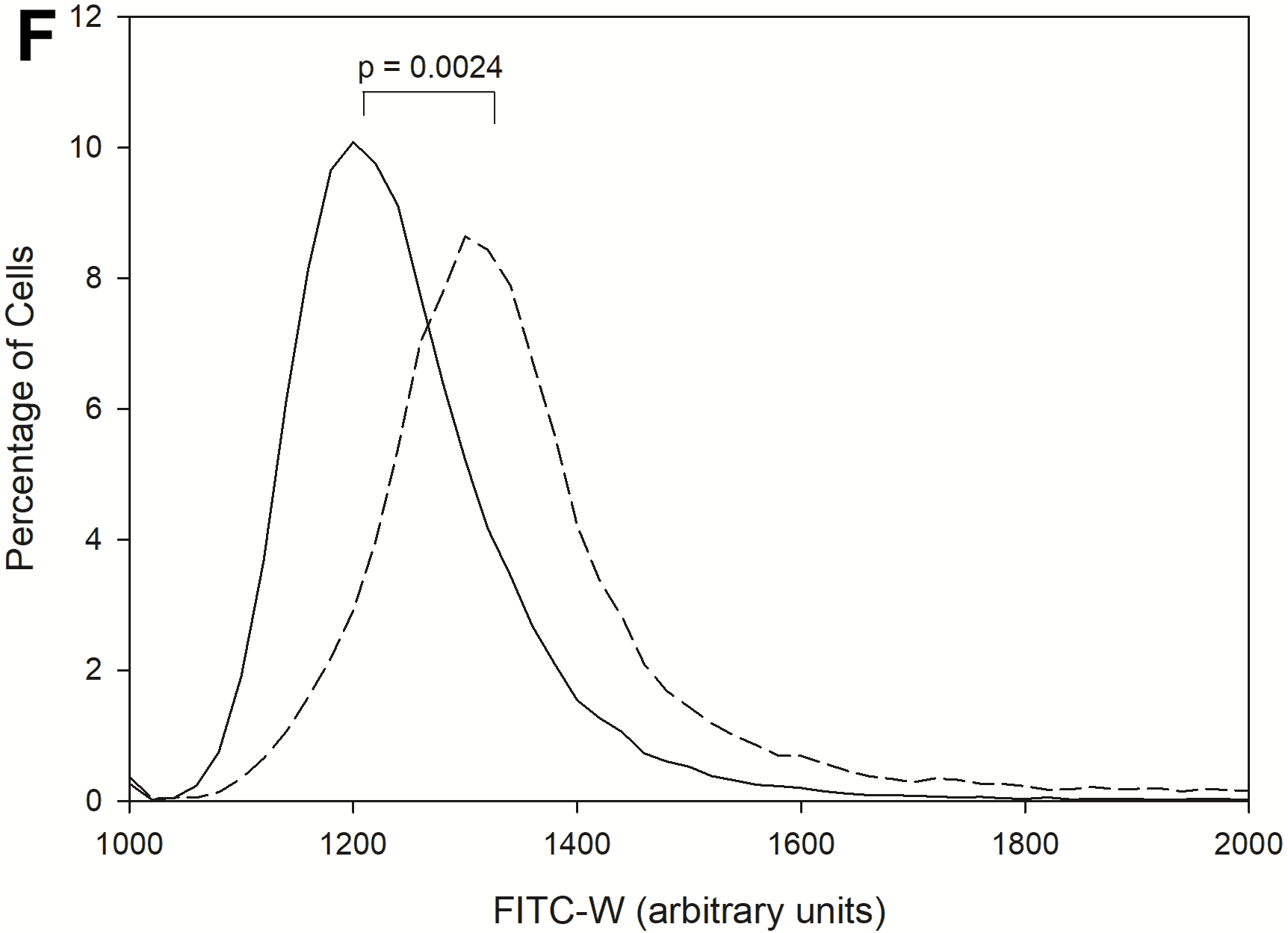


**FIGURE LEGENDS**

**Figure S1.** (A) SDS-PAGE analysis on 8% non-reducing gels showing the anti-CD38 mAb OKT10 and the IT OKT10-SAP. As shown here OKT10-SAP contains a mixture of hybrid molecules comprised of one antibody molecule covalently coupled to one (1:1), two (1:2) or three (1:3) saporin molecules. The identity of the band labelled 1:3 is uncertain and this may represent the presence of a 2:2 dimer conjugate. This IT mixture also contains small quantities of native OKT10 antibody (10%) and unconjugated saporin toxin (10%) as shown in (B).

**Figure S2.** The uptake of SAP-AF and OKSAP-AF into HSB-2 cells. (A) HSB-2 cells were incubated with SAP-AF or OKSAP-AF and live cell confocal images taken after 0, 2, 8 and 24 hours. The nucleus (red) was stained with Hoechst 33342. Co-localisation studies were performed between SAP-AF (green) and (B) the lysosomal marker LAMP-1 (red) or (C) the early endosomal marker EEA-1. Sites of co-localisation appear in yellow. The nucleus (blue) was stained with Hoechst 33342. Images presented are maximum projections of 21 x 1 µm Z-stacks. Scale bar represents 10 µm.

**Figure S3.** Pulse shape analysis of the uptake of SAP-AF and OKSAP-AF into HSB-2 cells. HSB-2 cells were incubated with SAP-AF or OKSAP-AF and analysed by flow cytometry after 0,2,8 and 24 hours. Dot plots showing FITC-H against FITC-W parameters are presented here demonstrating the reduction in FITC-W and increase in FITC-H over time. Each displayed dot plot represents approximately 10,000 events.

**Figure S4.** Endolysosomal escape of SAP-AF and OKSAP-AF into the cytosol. HSB-2 cells incubated with (A) SAP-AF or (B) OKSAP-AF for 24 hours were treated with 5µg/ml of SA and live cell confocal images taken after 0, 8, 16 and 24 hours. Untreated cells are shown for comparison. Endolysosomal escape is seen as a change from punctate vesicular fluorescence to a diffuse staining pattern throughout the cytoplasm. Images for the initial timepoint at zero hours are shown with and without Hoechst 33342 nuclear stain (red). Images presented are maximum projections of 21 x 1 µm Z-stacks. Scale bar represents 10 µm.

**Figure S5.** The use of pulse width analysis to investigate endolysosomal escape. Pulse width histograms show the distribution in FITC-W for HSB-2 cells from three combined experiments preincubated with SAP-AF for 24 hours before being treated with (A) 0.1 µg/ml, (B) 1 µg/ml or (C) 5 µg/ml of SA for a further 24 hours. Equivalent data for OKSAP-AF are shown in D, E and F respectively. Cells treated with SA are marked with a dashed line, each chart also shows the histogram for untreated cells, shown with a solid line. P values for the difference in median FITC-W between treated and untreated cells are shown on each chart (n=3 independent experiments).
